# Interface interaction of transition metal phthalocyanines with strontium titanate (100)

**DOI:** 10.3762/bjnano.12.39

**Published:** 2021-05-21

**Authors:** Reimer Karstens, Thomas Chassé, Heiko Peisert

**Affiliations:** 1 Institute of Physical and Theoretical Chemistry, University of Tübingen, Auf der Morgenstelle 18, 72076 Tübingen, Germany; 2 Center for Light–Matter Interaction, Sensors & Analytics (LISA+) at the University of Tübingen, Auf der Morgenstelle 18, 72076 Tübingen, Germany

**Keywords:** charge transfer at interfaces, strontium titanate, transition metal phthalocyanines

## Abstract

We study interface properties of CoPcF*_x_* and FePcF_x_ (*x* = 0 or 16) on niobium-doped SrTiO_3_(100) surfaces using mainly X-ray photoelectron spectroscopy and ultraviolet photoelectron spectroscopy. For all studied molecules, a rather complex, bidirectional charge transfer with the oxide substrate was observed, involving both the macrocycle and the central metal atom. For molecules of the first monolayer, an electron transfer to the central metal atom is concluded from transition metal 2p core level photoemission spectra. The number of interacting molecules in the first monolayer on the oxide surface depends on the central metal atom of the phthalocyanine, whereas the substrate preparation has minor influence on the interaction between CoPc and SrTiO_3_(100). Differences of the interaction mechanism to related TiO_2_ surfaces are discussed.

## Introduction

Interfaces between organic semiconductors and oxides are of increasing fundamental interest. Such interfaces determine key properties of a broad variety of electronic devices. Common examples are dye-sensitized solar cells, field-effect transistors (FETs), and sensors [1–2]. Possibly, one of the most extensively studied oxide material in this context is rutile titanium dioxide [3]. However, also interfaces between SrTiO_3_ (STO) and organic molecules are studied increasingly using both experimental [4–5] and theoretical approaches [6]. Possible applications of STO/organic interfaces include FETs [7–8], photodiodes [9], and organic spin valves[10].

Strontium titanate is a semiconductor with an indirect band gap of 3.25 eV [11] crystallizing in a perovskite structure with cubic unit cell. The conductivity can be increased by introducing oxygen vacancies into the crystal structure or by doping (e.g., n-type doping with niobium, Nb^5+^). Generally, two different terminations of STO(100) are known, that is, the surface can be either TiO_2_- or SrO-terminated. The TiO_2_ termination can be achieved by (ex situ) acid treatments [12–13] or water leaching [14–15]. The SrO termination is often achieved via thermal Sr segregation [16–18] or by deposition of SrO in vacuo [19–20]. Due to the thermal Sr segregation effect, sputtering and annealing procedures result commonly in SrO-terminated surfaces [21]. The detailed preparation procedures differ distinctly. For example, the temperature range for Sr segregation reaches from 570 K [22] to 1570 K [23]. In many cases, mixed surface terminations are obtained consisting of multiple islands of dominant terminations or SrO droplets [21–22,24–25].

**Figure 1:**
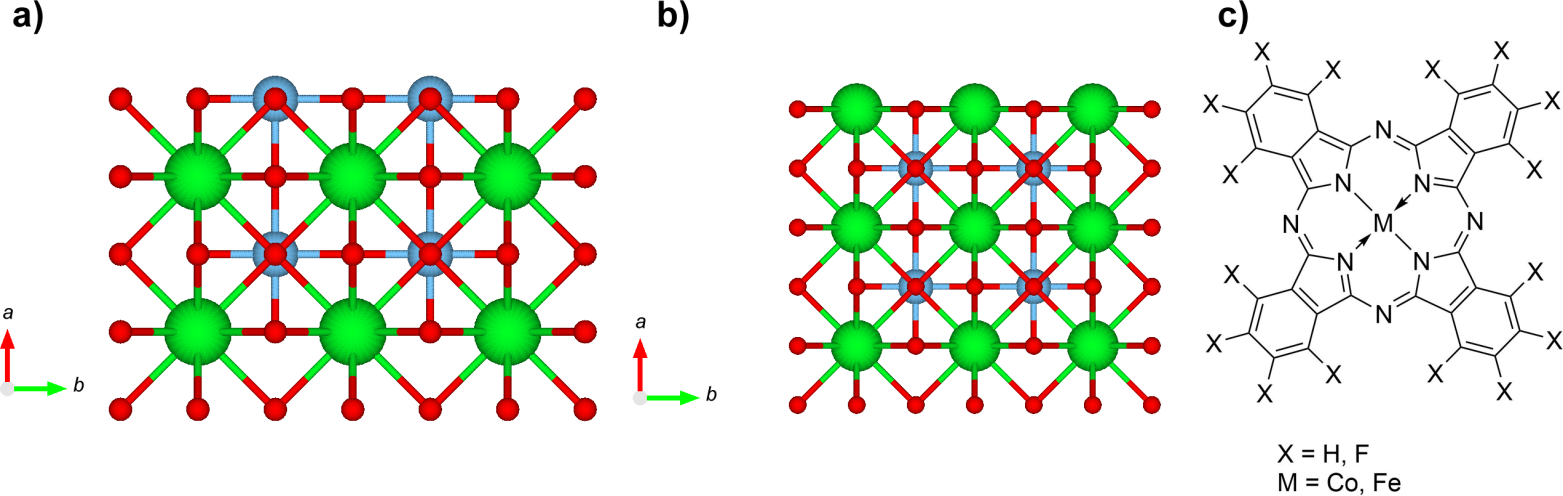
Bulk crystal structures (side views) of: (a) TiO_2_-terminated strontium titanate (100) and (b) SrO-terminated strontium titanate (100) (Ti^4+^: light blue, Sr^2+^: green, and O^2−^: red. The coordinate axes *a*, *b*, and *c* correspond, respectively, to the crystal orientations (100), (010), and (001)). (c) General chemical structure of transition metal phthalocyanines (TMPcs).

SrTiO_3_(100) bulk structures are visualized in Figure 1. LEED measurements (see below) gave no evidence for the appearance of occasionally discussed surface reconstructions after our preparation procedures [26–27]. In Figure 1a a TiO_2_-terminated surface is shown, where Ti^4+^ (light blue) and O^2−^ (red) ions form the outmost (top) layer. In contrast, the top layer of SrO-terminated STO in Figure 1b contains Sr^2+^ (green) and O^2−^ (red) ions, only. The length of the cubic lattice vectors is 3.91 Å [28] for both terminations. Due to the same symmetry of the surface structure, unfortunately, we cannot distinguish between different terminations by LEED. It might be expected that the presence of the different metal ions on the substrate surface (Sr or Ti) affects possible interactions with deposited organic molecules. We note that the surface structure in the topmost layer of TiO_2_-terminated STO significantly differs from most rutile TiO_2_ surfaces (e.g., (110) and (100)). In particular due to the presence of both Ti^4+^ and O^2−^ ions in the topmost layer of STO we may expect differences regarding molecule–oxide interactions.

The present study investigates interface interactions between different STO(100) surfaces and organic molecules. The general chemical structure of the chosen molecules is shown in Figure 1c. The electronic properties of transition metal phthalocyanines (TMPcs) can be widely chemically modified at both the central metal atom and the macrocycle. This chemical tunability allows for a broad variation of electronic properties. Hence, TMPcs are well suited for systematic studies of interface properties. The fluorination of TMPcs varies exceptionally the ionization potential (IP), affecting distinctly the interface properties [29–32]. As recently shown for FePcF*_x_*/MoS_2_ [33], a charge transfer at interfaces might be driven by the ionization potential difference between substrate and adsorbate. In addition, fluorination may affect significantly the adsorption geometry on surfaces as well as the single-crystal structure and arrangement in thin films [34–37]. Furthermore, local chemical interactions might become possible between particular atoms of substrate and adsorbate. For example, for many CoPc and CoPcF_16_ interfaces to noble metals, the interfacial interaction is governed by a local interaction between the Co 3d*_z_*_2_ orbital and states of the metal substrate [38–40]. Thus, the selected TMPcs allow for the study of both the influence of the ionization potential and the central metal atom on the properties of interfaces with differently prepared STO surfaces.

## Experimental

Niobium-doped strontium titanate (100) (Nb:STO) single crystals were purchased from MaTeck GmbH (Jülich, Germany; 10 × 10 × 0.5 mm^3^, 0.5 wt % Nb). The surfaces were typically prepared in vacuo by repeated cycles of Ar ion sputtering (0.5 kV, *p*(Ar) = 5 × 10^−5^ mbar, 30 min) and annealing (900 K, *p*(O_2_) = 4 × 10^−5^ mbar, 30 min). This method is called “preparation I” in the following. We note that this procedure results in a predominantly SrO-terminated, but mixed crystal surface. In order to modify the surface preparation, another procedure, called “preparation II”, was applied. After a wet chemical preparation, a monolayer of TiO_2_ was grown epitaxially on top of the STO surface by evaporation of Ti (0.6 Å) in an oxygen atmosphere (*p*(O_2_) = 4 × 10^−5^ mbar) at a substrate temperature of 910 K. The Ti thickness was monitored by the ion flux of the electron beam evaporator (EFM 3s, Focus GmbH) calibrated by a quartz microbalance. The estimation of the TiO_2_ thickness from the attenuation of the Sr 3d peak intensity supports the presence of an additional TiO_2_ layer on the STO substrate.

FePc (dye content 90%), CoPc and CoPcF_16_ were purchased from Sigma Aldrich Chemie GmbH (Steinheim, Germany), and FePcF_16_ was purchased from Synthon Chemicals GmbH & Co. KG (Bitterfeld-Wolfen, Germany). FePc and FePcF_16_ powders were resublimed before usage. The materials were evaporated from temperature-controlled crucibles. The nominal layer thickness was estimated from substrate- and adsorbate-related XPS intensity ratios using photoemission cross sections from Yeh and Lindau [41]. A nominal monolayer of lying molecules corresponds to a thickness of 0.34 nm estimated from the structure of an α-polymorph of TMPcs [42–44].

Photoelectron spectroscopy (PES) measurements were performed using an ultrahigh-vacuum setup equipped with a monochromatized standard source (Al Kα), a twin-anode standard source (Al Kα and Mg Kα), PHOIBOS 100 (X-ray photoelectron diffraction) or 150 (monochromatized X-ray photoelectron spectroscopy) hemispherical analyzers (SPECS), and a four-grid LEED optics (SpectaLEED, Omicron, Germany). The PES binding energy scale is calibrated to reproduce the binding energies of Cu 2p_3/2_ (932.6 eV), Ag 3d_5/2_ (368.2 eV), and Au 4f_7/2_ (84.0 eV). For X-ray photoelectron diffraction measurements, the angular acceptance of the analyzer was set to ±4°. Angular distribution curves were measured for the Sr 3d, Ti 2p, and O 1s photoemission peaks at two azimuths ([100] and [110], determined by LEED); the step width of the polar angle was 2°. The peak intensity was normalized by dividing the peak maximum by the background mean value at the lower binding energy side. PES spectra were analyzed using UNIFIT 2018 [45]. For the illustration of crystal structures the software VESTA [46] was used.

## Results and Discussion

### Characterization of the substrate surfaces

For related interfaces between rutile TiO_2_ and organic molecules, significantly different interaction strengths in similar systems were reported, which can be most likely ascribed to different substrate preparation procedures [47–50]. Therefore, we will apply two different, well-characterized preparation procedures. For preparation I, the STO single crystal was cleaned in vacuo as described above. Routinely, the cleanliness is checked by X-ray photoelectron spectroscopy and the quality of the surface structure by LEED. Examples of LEED images are shown in Supporting Information File 1 (Figure S1). It was reported that the work function ϕ_F_ correlates strongly with the termination of the STO substrate surface [19]. For SrO-terminated STO(100) ϕ_F_ is significantly lower (3.1–3.6 eV) than for TiO_2_-terminated STO(100) (4.5–4.8 eV). The work function, as determined from photoemission, was close to 3.9 eV for all STO single crystals prepared according to preparation I, indicating a mixed surface termination, likely with a slightly dominant contribution of SrO. In contrast, for preparation II, we obtained work functions between 3.93 eV (preparation II, experiment 2) and 4.15 eV (preparation II, experiment 1), although a very similar annealing procedure was applied. The work function variations indicate that different fractions of mixed terminations were prepared. The highest value of 4.15 eV points to a predominantly TiO_2_-terminated STO(100) surface. We assume that the final work function depends critically on the diffusion of Sr ions during the final annealing step and thus slightly different evaporation conditions or sample temperatures influence the surface composition distinctly. The different termination for samples with different work function is supported by the Ti/Sr/O ratios determined from overview spectra: We obtain Ti/Sr/O = 1:1.28:2.61 for the sample with ϕ_F_ = 4.15 eV and Ti/Sr/O = 1:1.45:2.80 for the sample with ϕ_F_ = 3.93 eV. Typical values for samples prepared according to preparation I are Ti/Sr/O = 1:1.46:2.85. We note that these values should be compared only relatively; absolute numbers are strongly influenced by photoelectron diffraction effects (see next paragraph).

Another method to analyze the termination of the STO(100) surface is X-ray photoelectron diffraction [26,51]. We measured angular distribution curves (ADCs) for Sr 3d, Ti 2p, and O 1s at two azimuths ([100] and [110]) with a variation of the polar angle between normal emission (0°) and 30° with a step width of 2°. Comparably sensitive to the surface termination are ADCs of the Sr 3d peak at the [100] azimuth [51–52]; examples are shown in Figure S2, Supporting Information File 1. While the different shape may generally indicate the presence of different surface structures, here, we obtain no unambiguous information about the actual surface termination, most likely due to the rather mixed termination already mentioned above. However, we can confirm the structural ordering of the near-surface region below the topmost layer, and we may rule out a completely SrO-terminated surface.

### CoPcF*_x_* on STO(100)

Possible interactions between organic molecules and different substrates may include charge transfer processes or even chemical reactions. In the case of semiconducting substrates, such a charge transfer would result in an interface doping of the substrate. Depending on the charge carrier concentration, the doping is accompanied by a shift of the Fermi level, visible as rigid energy shifts of all substrate-related core level spectra in photoemission.

**Figure 2:**
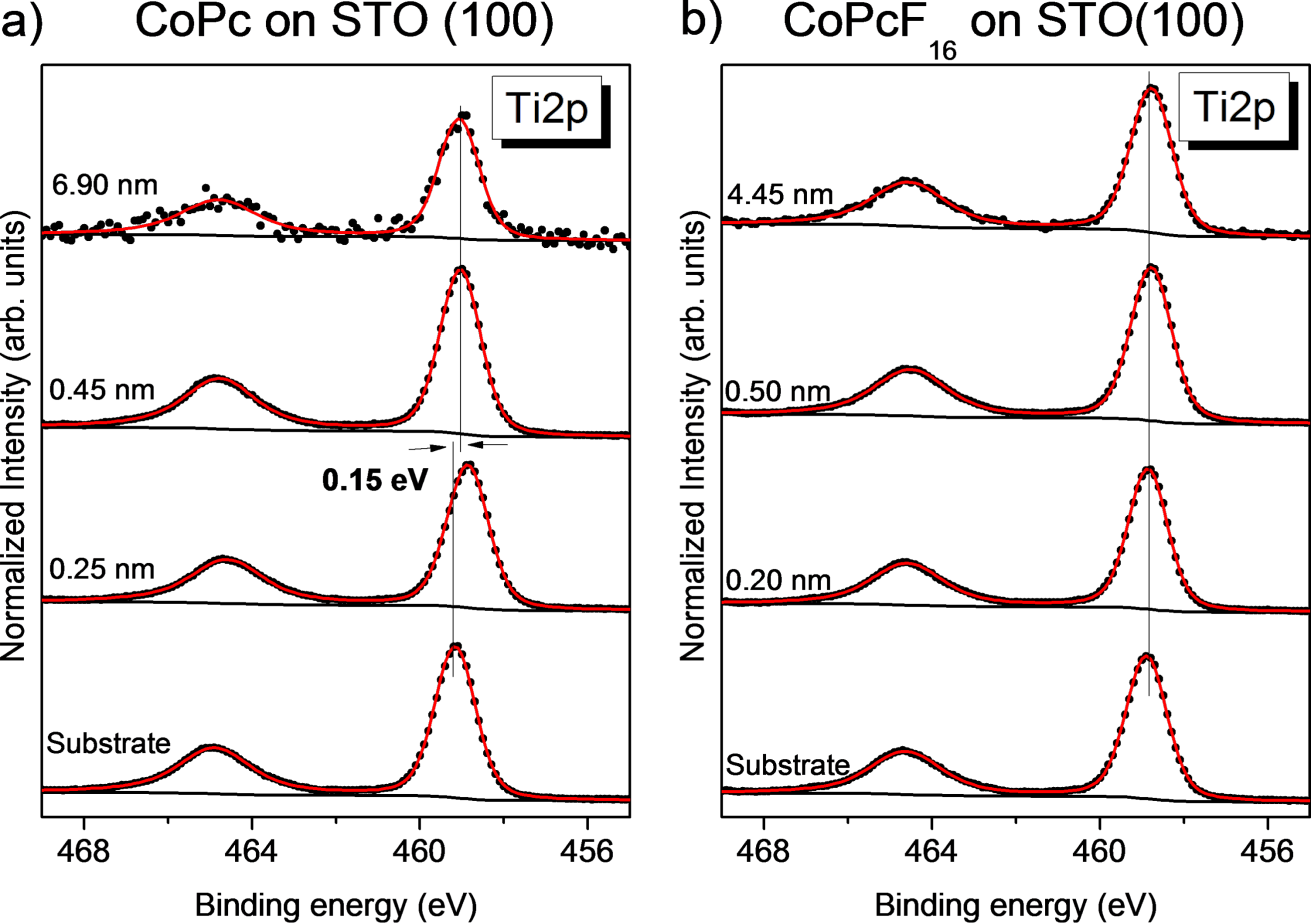
Development of Ti 2p spectra as an example for the change of substrate-related core level spectra with increasing thickness of (a) the CoPc or (b) the CoPcF_16_ films.

As an example, we discuss the development of Ti 2p spectra with increasing thickness of the organic overlayer (CoPc or CoPcF_16_) in Figure 2, Sr 3d and O 1s spectra are shown in Figure S3 and Figure S4 (Supporting Information File 1). All peak fit parameters are supplied in Supporting Information File 1 (Tables S1–S6).

All Ti 2p spectra in Figure 2 can be described by a single doublet arising from spin–orbit splitting, which can be assigned to Ti^4+^ ions of the substrate. The binding energy of the Ti 2p_3/2_ component of the pristine substrate is 459.15 eV (Figure 2a) or 458.9 eV (Figure 2b), indicating a slightly different position of the Fermi level in the gap of the STO semiconductor. Upon evaporation of the organic molecules, no changes of the peak shape can be detected, pointing to the absence of chemical interactions involving Ti atoms at the interface. However, we note that the surface sensitivity at the comparably high kinetic energies (mean free path of electrons about 2 nm) may hinder the unambiguous detection of different chemical states in the uppermost layer. Analogously, also for the other substrate-related O 1s and Sr 3d spectra (Figure S2 and Figure S3, Supporting Information File 1), no interface components were found. The small shift of the Ti 2p spectra in Figure 2a after deposition of CoPc to lower binding energies is also observed in the corresponding O 1s and Sr 3d spectra (Figure S2 and Figure S3, Supporting Information File 1) and might be assigned to an adsorbate-induced band bending at the interface (p-type doping).

**Figure 3:**
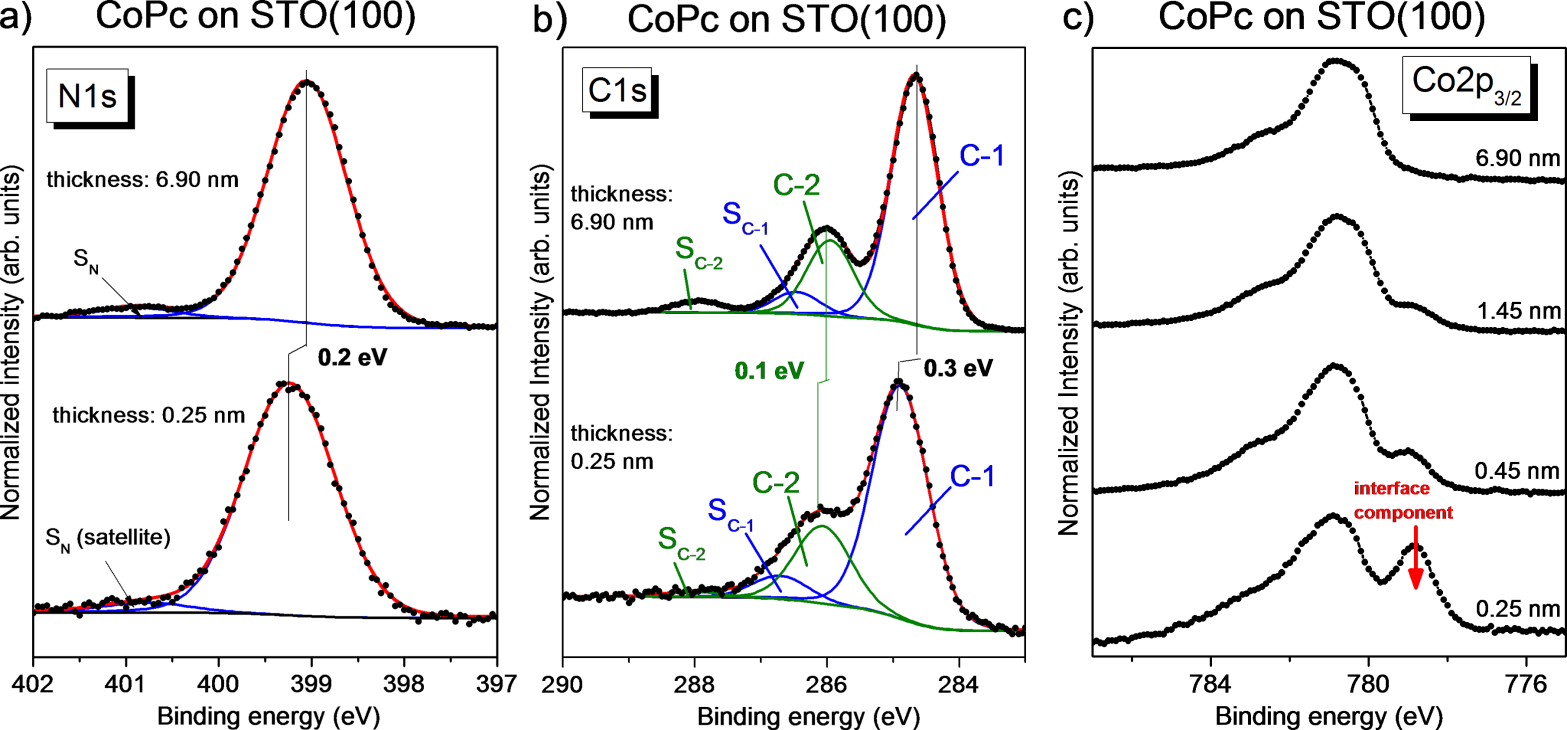
CoPc on STO(100). (a) N 1s, (b) C 1s, and (c) Co 2p core level spectra for a multilayer film compared to a coverage of a monolayer. The Co 2p spectra show clearly an interface component at lower binding energy than the main component (see arrow).

Adsorbate-related core level spectra are shown in Figure 3 for different film thicknesses. For the 0.25 nm and the 0.45 nm film, the Sr 3p_1/2_ background was subtracted in the C 1s spectra. Original data and the background procedure are shown in Figure S5 (Supporting Information File 1). The peak fit parameters are given in Table S7 and Table S8 (Supporting Information File 1).

For the thickest films in Figure 3 (6.9 nm) the molecule-related core level spectra can be described analogously to the literature of transition metal phthalocyanines [53–55]. A single main component was assumed for N 1s, due to the typically small energy separation of the two chemically inequivalent nitrogen atoms (0.3 eV for FePc [56]). For C 1s, we distinguish between aromatic carbon atoms of the benzene rings (C-1) and pyrrole carbon atoms linked to nitrogen (C-2). All main components in the N 1s and C 1s spectra are accompanied by satellite components at 1.6–1.8 eV higher binding energy (S_N_, S_C-1_, and S_C-2_). The shape of the Co 2p_3/2_ spectrum is characterized by complex multiplet structures of the Co(II) ion with d^7^ electron configuration [39,55,57–58].

The monolayer spectra (0.25 nm) of the macrocycle (N 1s and C 1s) in Figure 3 can be essentially described by the model applied for the bulk-like thicker films. For both N 1s and C 1s spectra, a slight broadening is observed for monolayer spectra compared to the bulk. The Gaussian width increases from 1.0 to 1.1 eV and from 0.8 to 0.9 eV for N 1s and C 1s, respectively. Such a broadening might be ascribed to adsorption at inequivalent adsorption sites or other kinds of disorder, which may result in a statistical distribution of orbital energies [59]. Also visible is a shift of the monolayer N 1s and C 1s core level spectra to 0.2–0.3 eV higher binding energies with respect to the 6.9 nm film in Figure 3. Many reasons, including charge transfer and a different screening of the photohole due to a different environment, may cause such shifts. The shift to higher binding energy at the substrate interface is rather unlikely for screening and might therefore point to a charge transfer from the macrocycle of the molecule to the substrate. This does generally not contradict the abovementioned p-type doping of the substrate surface, which is connected to the total interface charge redistribution, because our data indicate also a substantial opposite charge transfer towards the Co central metal atoms of the molecules in the first monolayer (see below). The different shifts for N 1s and C 1s components point to a different charge distribution for the monolayer compared to the thick film, in good agreement to CoPcF_16_ on STO discussed below.

Most visible are thickness-dependent changes of the shape of Co 2p_3/2_ spectra in Figure 3c. At the low binding energy side of the main structure at about 780.9 eV, a new peak develops with decreasing film thickness. The binding energy difference of about 2 eV reminds of interface interactions observed at both metallic and oxidic substrates [39–40,58,60–62]. The lower binding energy of the interface component arises from an electron transfer to the central Co ion of CoPc, which can be accompanied by a backdonation from the macrocycle to the substrate [61–62], in good agreement to the observed binding energy shifts of N 1s and C 1s core level spectra discussed above. The remaining intensity of the main component at the lowest coverage of 0.25 nm in Figure 3c indicates that not all molecules of the first layer are involved in the interaction. In other words, the strength of the interface interaction depends on the particular adsorption site.

**Figure 4:**
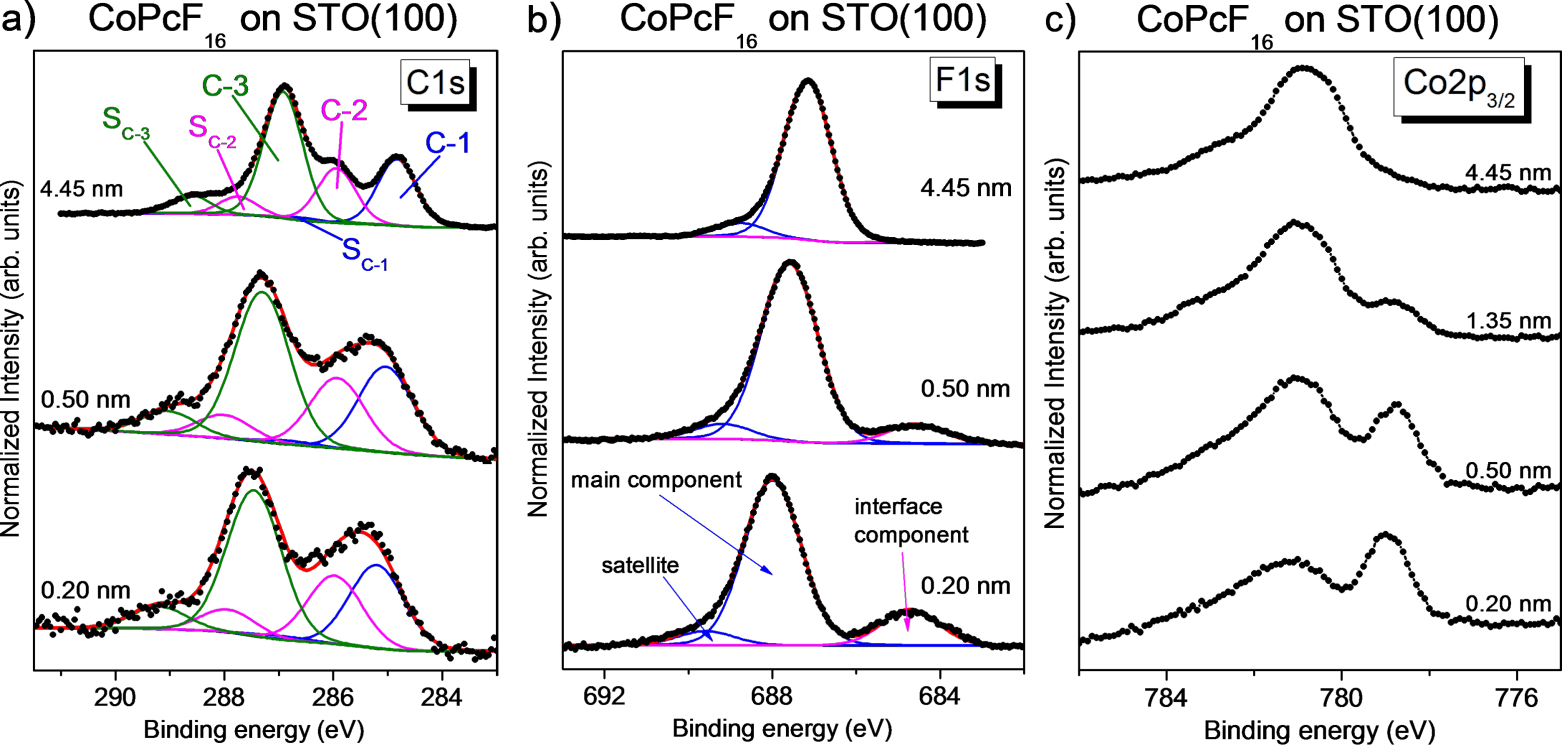
CoPcF_16_ on STO(100). Thickness-dependent core level spectra: (a) C 1s, (b) F 1s, and (c) Co 2p_3/2_. Distinct interface components are visible in the F 1s and Co 2p_3/2_ spectra.

The question arises whether or not the interaction is further affected by the fluorination of the CoPc molecule. C 1s, F 1s and Co 2p_3/2_ core level spectra of CoPcF_16_ on STO are shown in Figure 4. The peak fit data are given in Table S9 and Table S10 (Supporting Information File 1). The corresponding N 1s core level spectra are similar to those of CoPc; they are also shown in Figure S6 (Supporting Information File 1). Analogously to CoPc, the core level spectra can be well described using models from the literature. As for CuPcF_16_ [29], in the C 1s core level spectra, besides the components C-1 and C-2 in the CoPc C 1s spectra, an additional component (C-3) accompanied by a satellite peak has to be introduced. The peak fit of the bulk-like, thickest film reproduces well the stoichiometric composition (see Tables S9–S11, Supporting Information File 1). Single components and a satellite were used for fitting the N 1s and F 1s spectra. The multiplet structure of the Co 2p_3/2_ spectrum is similar to that of CoPc (Figure 3).

Similar to the case of CoPc, core level spectra related to the macrocycle are broadened at low coverages, possibly due to different adsorption sites on the substrate lattice. Also, distinct shifts to higher binding energies can be detected with decreasing film thickness. Trends for binding energy shifts of the C-1 component as a function of the CoPc (and FePc) thickness are almost independent on the substrate preparation, as summarized in Figure S7 (Supporting Information File 1). A closer look at the peak positions summarized in Table 1 reveals that the shifts are not the same for each component; they range from 0.1 to 0.8 eV. The different peak shifts indicate a different charge distribution for molecules of the first monolayer and thus point to a rather complex, bidirectional charge transfer involving the macrocycle of CoPcF_16_.

**Table 1:** Binding energy shifts of core level spectra of CoPcF_16_ as a function of the film thickness.

	C-1	C-2	C-3	N 1s	F 1s

4.45 nm	284.8	285.9	288.9	398.9	687.2
0.2 nm	285.2	286.0	289.3	399.4	688.0
Δ*E*	0.4	0.1	0.4	0.5	0.8

In addition, a distinct interface component is visible in the F 1s spectra of Figure 4b at low coverages. The low binding energy of 684.7 eV points to the formation of metal–fluorine bonds (e.g., Ti–F) [63–64]. Thus, we assume that some of the C–F bonds of the CoPcF_16_ molecules are broken at the interface, most likely at more reactive sites only. This is qualitatively in good agreement with the smaller relative intensity of the C–F bonding related to the C-3 component in the corresponding C 1s spectra at the interface (cf. Table S9, Supporting Information File 1). From the F 1s peak fit we conclude an average of two to three broken C–F bonds per molecule (Table S10, Supporting Information File 1). We note that on defect-rich, highly reactive TiO_2_(100) a similar effect has been observed [50]. It might be expected that the breaking of C–F bonds will significantly affect electronic interface properties. The bond breaking might be supported by the higher electronegativity difference between Ti or Sr and fluorine compared to C–F and the typically high Ti–F and Sr–F bond energies. Indeed, a substrate-induced decomposition of the C–F bond was observed for perfluoropentacene on coinage metals [65]

Another interface component is found in the Co 2p_3/2_ spectra of Figure 4. Similar to CoPc on STO, the interface component appears at about 2 eV lower binding energy (779.0 eV) compared to the main component of the thickest film (780.9 eV). Thus, analogously to CoPc, the interface component in the Co 2p_3/2_ spectra can be understood by an electron transfer from the substrate to the Co ion of CoPcF_16_. The reduction of the Co ion results in a significant change of the multiplet structure arising from final-state effects due to the overlap of Co 2p and Co 3d wave functions. The shape of satellite structures above 780 eV for the reduced Co ion in CoPc is discussed for the CoPc/Ag interface in [58]. Since satellite features in Figure 4c appear in the same energy range as the main features of the bulk-related spectrum, an exact determination of the relative amount of reduced Co atoms is difficult; we estimate 60–80%. In addition, this hinders a discussion of thickness-dependent energy shifts of the Co 2p spectra.

Since such a charge transfer is not observed for CoPc on oxygen-terminated TiO_2_ surfaces [49,60], one might argue that a local interaction occurs between the metal atoms of the STO terminal layer and the Co ions of CoPc or CoPcF_16_. The different intensity of the interface component in the Co 2p_3/2_ spectra for both molecules may indicate a different arrangement of the molecules on the STO(100) surface due to the different size of the molecules and/or different intermolecular interactions. A strong repulsion is expected for CoPcF_16_ with its negatively charged outer fluorine atoms. Further, for CoPcF_16_, adsorption sites might be triggered by the observed C–F bond dissociation. However, uncertainties in the estimation of the monolayer thickness from XPS intensities only cannot be ruled out completely.

### FePcF*_x_* on STO(100)

To investigate whether the observed local interaction between the central metal atom of CoPcF*_x_* (*x* = 0 or 16) and the STO substrate is specific for the Co ion, we study interface properties of the related system FePcF*_x_* on STO(100). Similar to CoPcF*_x_* on STO(100), the substrate-related core level spectra (Supporting Information File 1, Figure S8 and Figure S9) do not show changes of the peak shape upon FePc deposition, that is, they are not sensitive enough to reveal possible interactions of the topmost layer. Only for FePcF_16_ on STO (Supporting Information File 1, Figure S9), energy shifts towards 0.2–0.3 eV lower binding energies upon FePcF_16_ deposition can be detected, which may indicate an adsorbate-induced band bending (p-type doping, electron transfer from STO to FePcF_16_).

**Figure 5:**
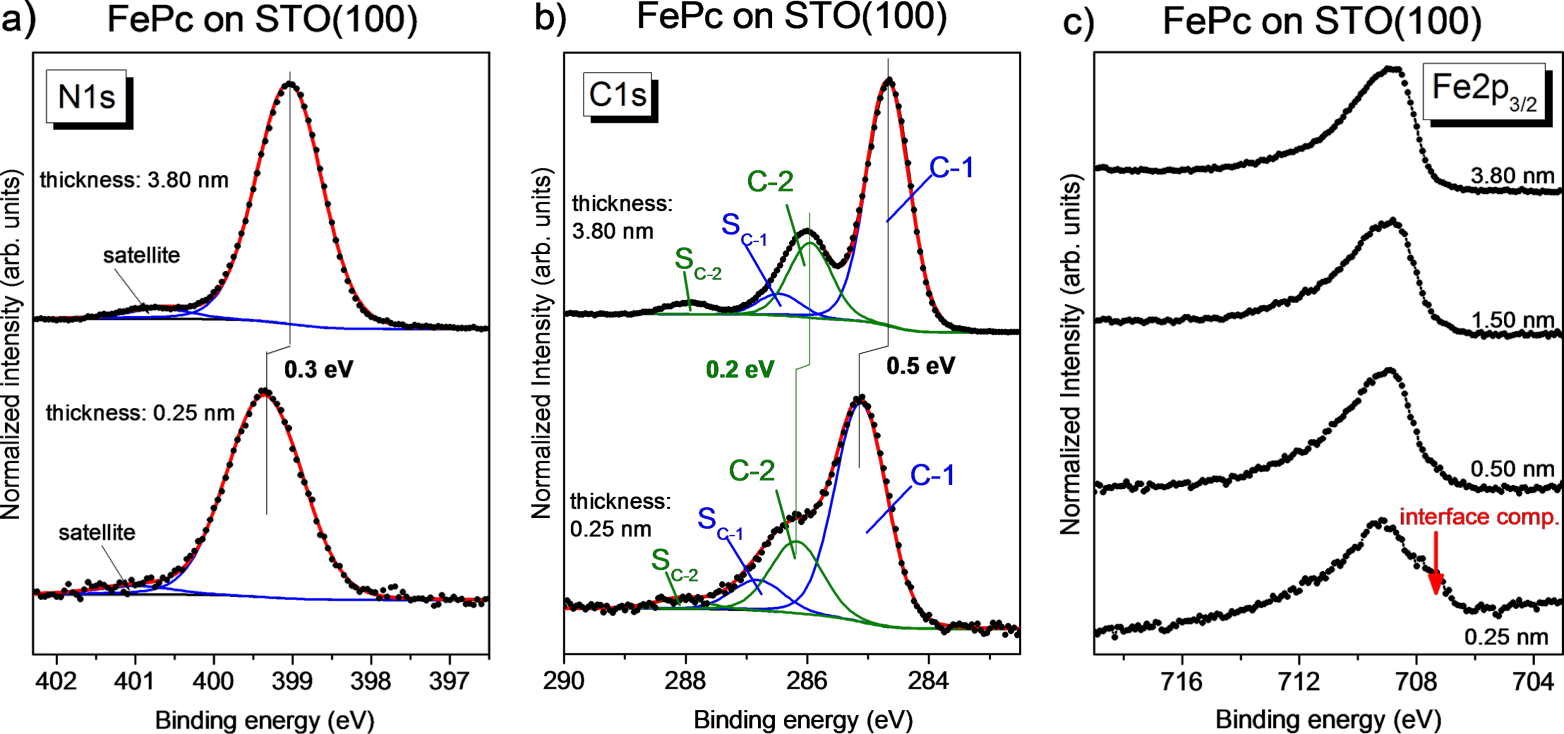
FePc on STO(100). (a) N 1s, (b) C 1s, and (c) Fe 2p core level spectra of multilayer films compared to a coverage of about a monolayer. The Fe 2p spectra show clearly an interface component at the lower binding energy side of the main peak (see arrow).

The macrocycle-related N 1s and C 1s core level spectra of FePc (Figure 5a,b) exhibit a thickness dependence, very similar to that for CoPc (cf. Figure 3). A broadening of monolayer spectra is observed, which can be explained by different environments or adsorption sites on the substrate. In addition, different energy shifts for each component are visible, most likely due to a different charge distribution for molecules of the first monolayer compared to the bulk-like, thicker film.

The FePc spectrum of the thicker film in Figure 5c exhibits the typical multiplet structure known for Fe^2+^ in FePc [56,66–67] with the intensity maximum at a binding energy of 708.8 eV. With decreasing layer thickness, a shoulder at the low binding energy side develops, which can be assigned to an interface peak (red arrow in Figure 5c). The lower binding energy compared to the main component indicates an electron transfer from the substrate to the Fe ion of FePc. For the lowest thickness of 0.25 nm (less than one monolayer), the interface component at a binding energy of 707.7 eV is not dominant in the spectrum; most intensity is found in the range of bulk-related main structures (shifted to 0.4 eV higher binding energy compared to the bulk). This reveals that not all FePc molecules interact with the STO substrate in the same manner. The estimation of the ratio between interacting/non-interacting molecules is hindered due to lack of a (reference) multiplet spectrum of reduced Fe ions in FePc. However, comparing Figure 3c and Figure 5c, it seems to be evident that the number of interacting FePc molecules is smaller than the number of interacting CoPc molecules on STO. This may point to a different adsorption geometry, where the number of molecules adsorbed with the central metal atom at a reactive substrate site is different and the probability is higher in the case of CoPc. A clear template effect of anisotropic substrates is also observed for related strong interacting interfaces [68]. Alternatively, one might speculate that the Co ion of CoPc is able to interact also with less reactive sites.

**Figure 6:**
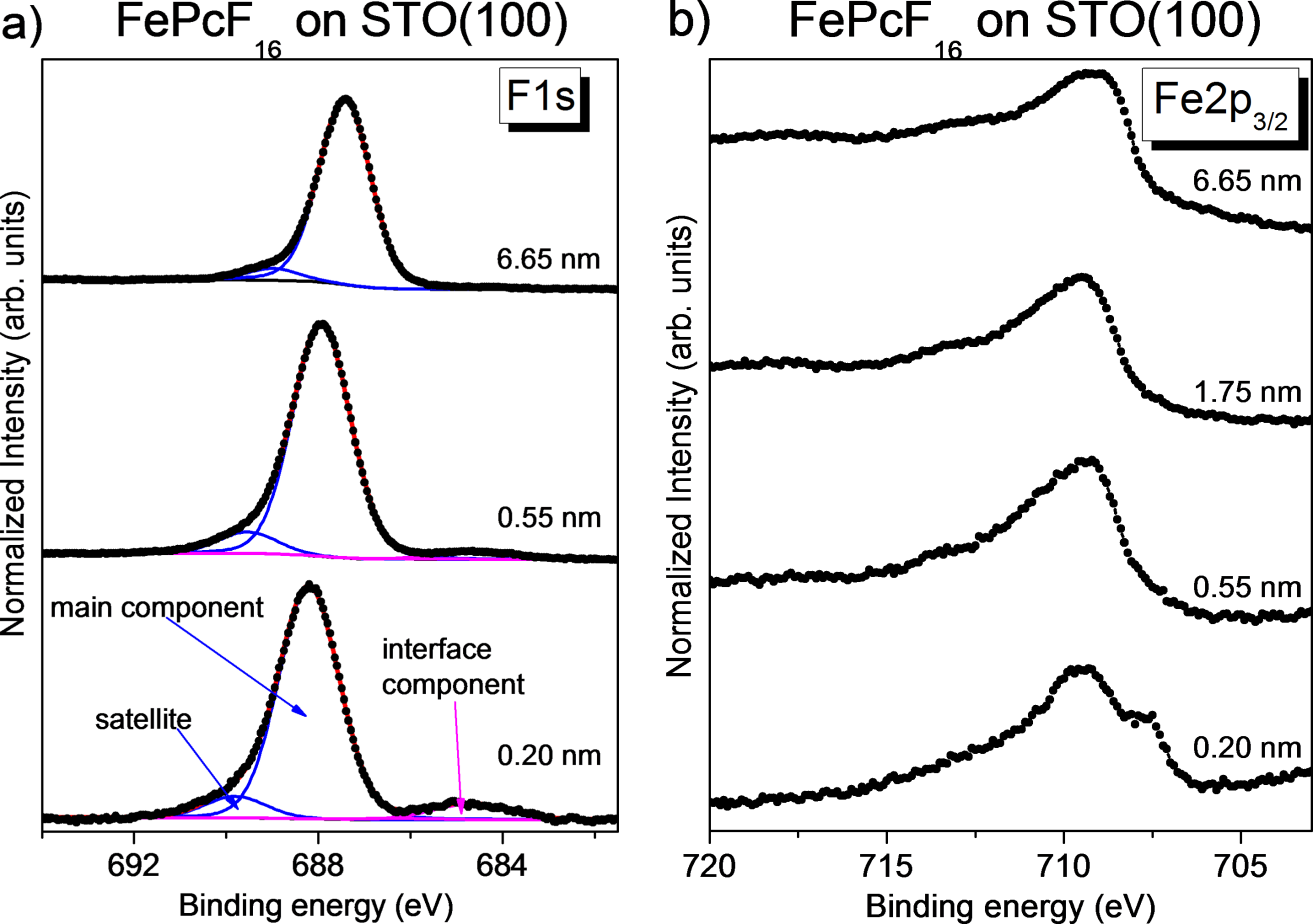
FePcF_16_ on STO(100). Thickness-dependent F 1s (a) and Fe 2p_3/2_ (b) core level spectra. The interface component in the Fe 2p_3/2_ spectra (see arrow) is similar to that in the FePc spectra (Figure 5).

Also for FePcF_16_, evidence for interactions with the STO(100) substrate can be detected in the adsorbate-related core level spectra. C 1s and N 1s spectra (Figure S10, Supporting Information File 1) as well as F 1s spectra (Figure 6a) show (different) shifts to higher binding energies for the monolayer, most likely due to charge transfer from the macrocycle to the substrate. Since the interface component in the Fe 2p_3/2_ spectra (Figure 6b) and also the substrate-related core level shifts (Figure S9, Supporting Information File 1) point to an opposite electron transfer, the interactions are rather complex and bidirectional.

Considering CoPcF_16_ on STO (cf. Figure 4b), the F 1s spectrum of the monolayer in Figure 6a exhibits an additional interface component. However, the intensity of the interface peak is distinctly smaller (8% of the main peak, corresponding to one to two broken C–F bonds per molecule) compared to CoPcF_16_. This might point to a different adsorption geometry for both molecules, where the probability of the location of C–F bonds at reactive substrate sites is different. The intensity of the interface component in the Fe 2p_3/2_ spectra in Figure 6c is comparable to that of FePc on STO. This indicates that for a comparable number of molecules of the first layer the central Fe ion interacts with the substrate surface. We note that the shape of the Fe 2p_3/2_ spectrum of the thick FePcF_16_ film in Figure 6b is not exactly comparable to the spectrum of the thickest FePc film in Figure 5c, which might be due to a superposition with intense high-energy-loss structures from F 1s photoemission (at about 688 eV) or a possible different spin state of Fe in both molecules [69].

In summary, there are no general differences in the interface properties of FePc and FePcF_16_ on STO(100). In both cases, for a significant number of molecules in the first monolayer, an electron transfer to the central Fe ion occurs, whereas an opposite charge transfer is proposed for the macrocycle. In addition, the interface interaction does not solely depend on the transition metal center, there are many similarities for CoPc/FePc and CoPcF_16_/FePcF_16_ on STO(100).

### Influence of the STO(100) preparation on interface interactions

We will study the influence of the substrate preparation on the interface properties at the example of CoPc. Substrates were alternatively prepared according to preparation II described in the Experimental section, where the last preparation step was the evaporation of Ti under oxygen partial pressure followed by annealing. Two experiments were performed using substrates with slightly different work functions (experiment 1: ϕ_F_ = 4.15 eV and experiment 2: ϕ_F_ = 3.93 eV). Substrate-related core level spectra show (similar to preparation I) no significant changes upon CoPc deposition for both experiments (Figure S11 and Figure S12, Supporting Information File 1). Also, the thickness dependences of macrocycle-related N 1s and C 1s core level spectra (Figure S13 and S14, Supporting Information File 1) are very similar to those of CoPc on STO(100) prepared according to preparation I.

**Figure 7:**
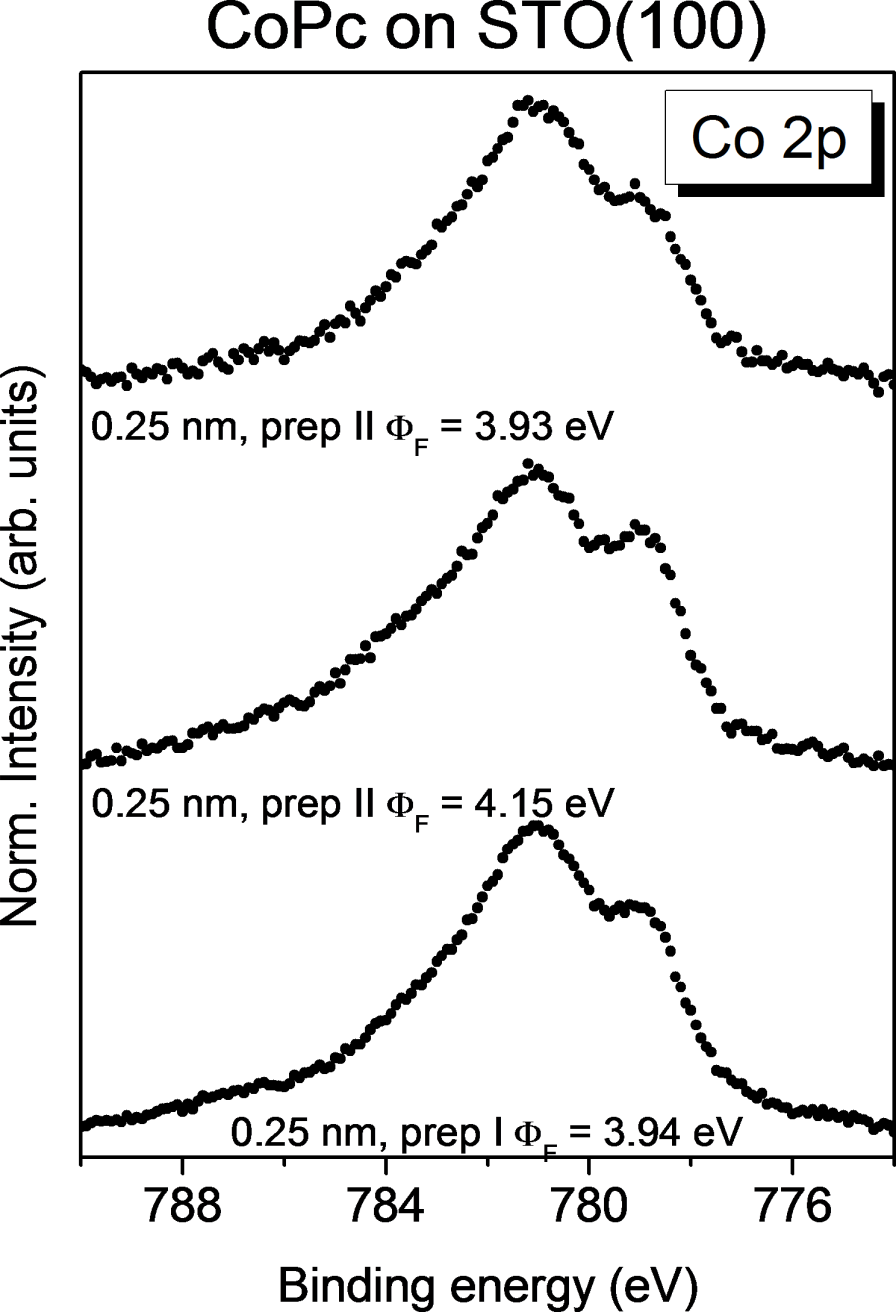
CoPc on STO(100). Co 2p_3/2_ core level spectra for a monolayer of CoPc deposited on differently prepared STO surfaces.

Small differences might be visible in the intensity of the interface component in Co 2p spectra for monolayer coverage of CoPc on differently prepared STO(100) surfaces (Figure 7). The complete thickness-dependent series for CoPc on STO(100) prepared according preparation II are given in Figure S15 (Supporting Information File 1). The spectrum of CoPc on STO (preparation I) is comparable to that in Figure 3c. For better comparability, it was taken from another experiment measured using a non-monochromatized X-ray source. It seems that the relative intensity of the interface component is somewhat higher for CoPc on the STO substrate with the highest work function (Figure 7, middle) compared to the other two substrate preparations. The reason might be a different surface termination; but slightly different preparation conditions may affect also the surface morphology and roughness. Notably, for all three preparations the intensity of the interface component is in the range between 40% and 60%. That is, only for about the half of the molecules an interaction between the Co ion and the STO(100) substrate is visible.

Thus, in our case, the preparation seems to have little influence on the interaction between CoPc and STO(100). We emphasize, however, that we obtained a mixed substrate termination for all three preparation procedures.

## Conclusion

We studied interface properties of CoPcF*_x_* and FePcF*_x_* (*x* = 0 or 16) on niobium-doped STO(100) surfaces. For all molecules, different shifts of core level photoemission peaks with overlayer thickness were observed, indicating a charge transfer from the macrocycle to the substrate. For a distinct number of molecules of the first monolayer (30–80%), an opposite electron transfer to the central metal atom was concluded from transition metal 2p spectra. Thus, the results point to a rather complex, bidirectional charge transfer involving both the macrocycle and the central metal atom of the studied molecules. In addition, for fluorinated molecules, the breaking of some (up to three per molecule) C–F bonds was observed. The number of interacting molecules depends on the central metal atom (CoPcF*_x_* > FePcF*_x_*), most likely due to different adsorption geometries or a different ability to interact with less reactive adsorption sites of the substrate. Additionally, for the example of CoPc on STO(100) it was shown that the applied substrate preparation procedures have little influence on interface properties.

We emphasize, that the interaction mechanism is very different to related rutile TiO_2_ surfaces, where strong local interactions via the nitrogen atoms were observed [47,50]. In contrast, interactions via the central metal atom are hardly observed on rutile TiO_2_ [50]. A reason for the different behavior of rutile and STO(100) surfaces might be the different composition of the outermost surface layer. For rutile TiO_2_ surfaces the topmost layer consists of oxygen ions, whereas for STO(100), depending on the termination, Ti or Sr atoms are also present on the surface. The STO(100) surfaces prepared in this study exhibit a mixed surface termination. Consequently, the interface interaction between the central metal atom of a part of the TMPcF*_x_* molecules in the first monolayer might be related to local interactions with Ti and/or Sr atoms. Also, interactions between the macrocycle of the TMPc and the rutile TiO_2_ surfaces seem to depend critically on the number of surface defects [50]. Thus, the absence of interface components (e.g., in N 1s spectra) for the TMPcs on STO(100) may point to a comparably defect-free surface. The cleavage of some intramolecular C–F bonds of TMPcF_16_ was observed at both STO(100) and defect-rich rutile surfaces. Since not all C–F bonds are broken, this reaction occurs only at particular sites. It seems that, generally, such oxidic surfaces are more prone to support the cleavage of intramolecular C–F bonds in comparison to metal surfaces.

## Supporting Information

File 1 Additional experimental data.
