# Supplementary material for: Interface interaction of transition metal phthalocyanines with strontium titanate (100)
Source: Beilstein J Nanotechnol. 2021 May 21;12:485–96. doi: 10.3762/bjnano.12.39 (PMC8144905; doi:10.3762/bjnano.12.39)
Supplement: File 1 — Additional experimental data. [file Beilstein_J_Nanotechnol-12-485-s001.pdf]

BEILSTEIN JOURNAL OF NANOTECHNOLOGY

Supporting Information

for

Interface interaction of transition metal phthalocyanines with strontium titanate (100)

Reimer Karstens, Thomas Chassé and Heiko Peisert

Beilstein J. Nanotechnol. **2021,** *12,* 485–496. doi:10.3762/bjnano.12.39

Additional experimental data

License and Terms: This is a supporting information file under the terms of the Creative Commons Attribution License (https://creativecommons.org/licenses/by/4.0). Please note that the reuse, redistribution and reproduction in particular requires that the author(s) and source are credited and that individual graphics may be subject to special legal provisions.
The license is subject to the *Beilstein Journal of Nanotechnology* terms and conditions: (https://www.beilstein-journals.org/bjnano/terms)

Content

Figure S1: LEED 1x1 pattern of one exemplary STO(100) single crystal (preparation I).

Figure S2: Sr 3d angular distribution curves at the [100] azimuth for different substrate preparations.

Figure S3: STO(100)-related core level spectra and their development with increasing CoPc film thickness.

Figure S4: STO(100)-related core level spectra and their development with increasing $CoPcF_{16}$ film thickness.

Figure S5: CoPc on STO(100): C 1s core level spectra and their modification for the subtraction of the Sr 3p signal from the C 1s peak for the interface layers of the organic molecules.

Figure S6: $CoPcF_{16}$ on STO(100): N 1s core level spectra.

Figure S7: Shifts of the binding energy of the C 1s C-1 component for the different molecules and preparations.

Figure S8: STO(100)-related core level spectra and their development with increasing FePc film thickness.

Figure S9: STO(100)-related core level spectra and their development with increasing $FePcF_{16}$ film thickness.

Figure S10: $FePcF_{16}$ on STO(100).

Figure S11: Substrate spectra of experiment 1 (preparation II).

Figure S12: Substrate spectra of experiment 2 (preparation II).

Figure S13: Organic spectra of thinnest and thickest film of experiment 1 (preparation II).

Figure S14: Organic spectra of thinnest and thickest film of experiment 2 (preparation II).

Figure S15: Comparing $Co2p_{3/2}$ spectra from experiment 1 (Φ_F = 4.15 eV) and 2 (Φ_F = 3.93 eV);

Tables S1–S32: Peak fit parameters for the spectra shown in the main manuscript.

Substrate surface quality after preparation

a) b)

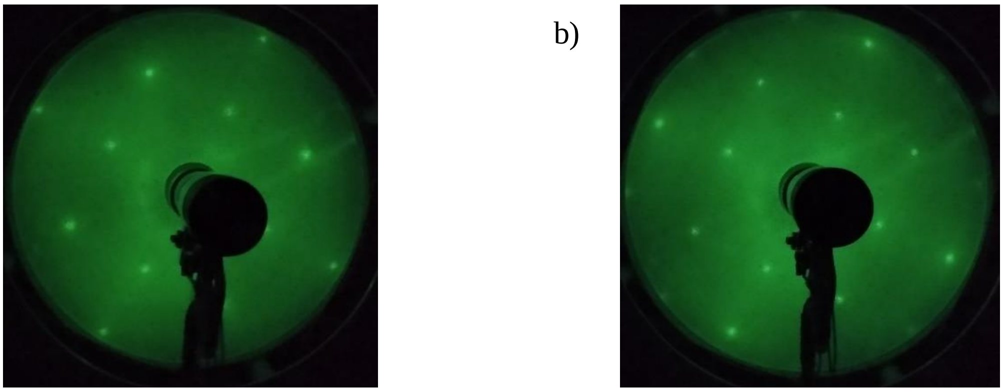

Figure S1: LEED 1 × 1 pattern of one exemplary STO(100) single crystal (preparation I) detected with: (a) 80 eV and (b) 95 eV. The pictures are showing clear spots in a typical cubic pattern with no hint for a superstructure and a weak background, so they attest a smooth substrate surface.

Determination of the surface termination from ADCs

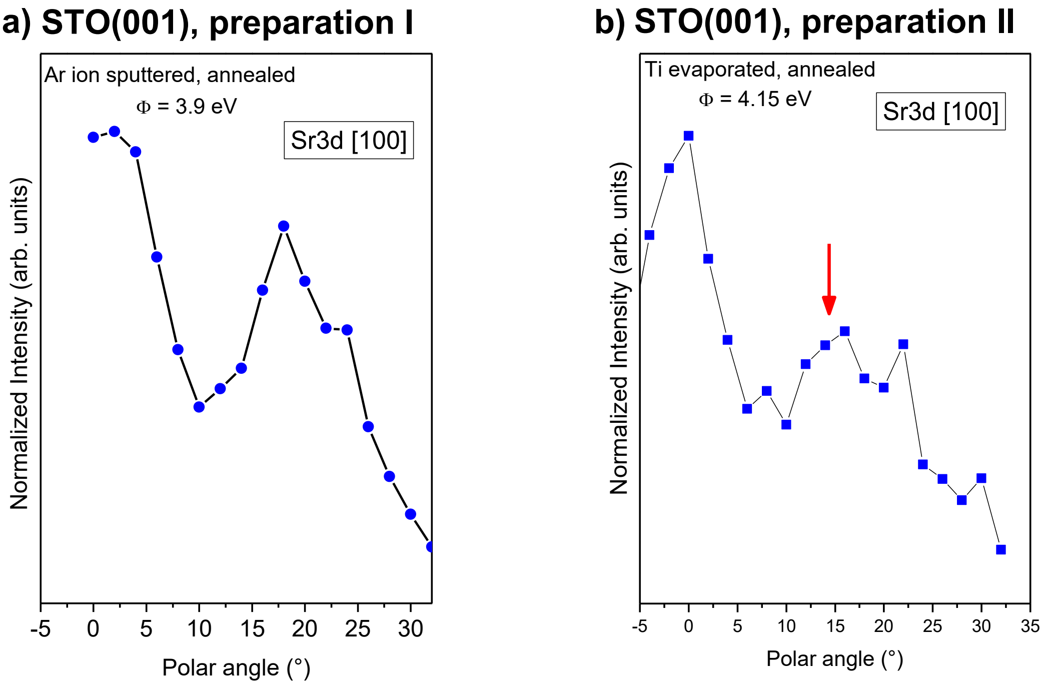

Figure S2: Sr 3d angular distribution curves at the [100] azimuth for different substrate preparations. Both ADCs exhibit the global maximum at an angle of 0°, followed by a local maximum at an angle of about 17° and a shoulder at around 23°. The local maximum at 17° (see arrow) might be sensitive on the surface termination and is expected to be stronger for TiO_2-terminated surfaces [1]. This is not the case for our preparation, most likely due to a strong effect of non-surface emitters on the photoelectron diffraction signal, as recently reported [2]. Another reason could be the presence of strontium oxide islands on a different termination, causing, among others, shadowing effects. However, the different ADCs indicate the presence of different surface structures after both preparation procedures.

Substrate spectra and peak fit parameters for CoPc on STO(100) prepared by preparation I

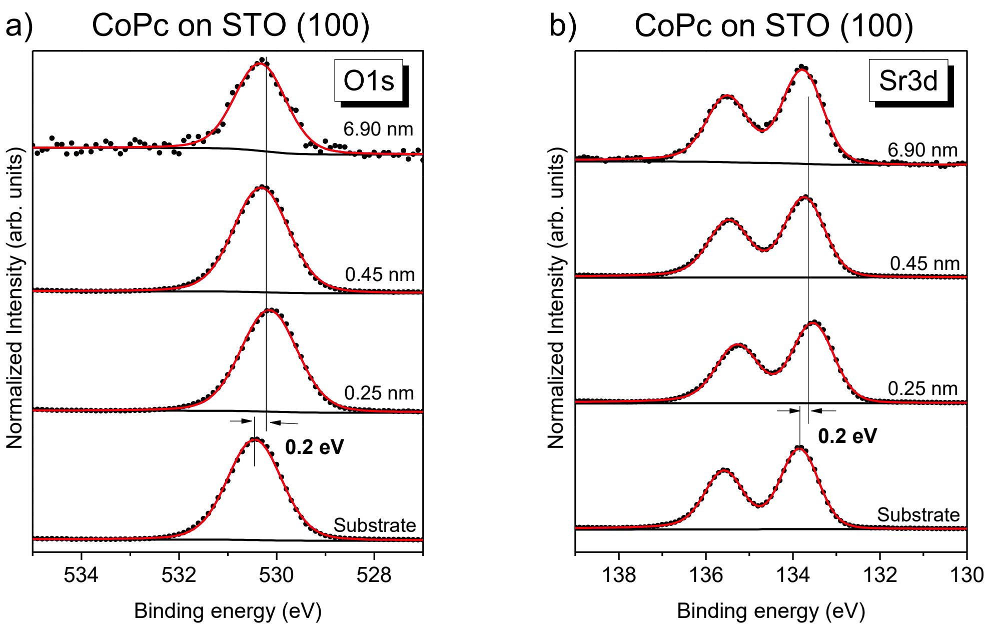

Figure S3: STO(100)-related core level spectra and their development with increasing CoPc film thickness. (a) O 1s and (b) Sr 3d. Small energy shifts may point to doping as a result of the CoPc adsorption. All spectra ca be fitted using a single singlet (O 1s) or doublet (Sr 3d) peak.

Table S1: Ti 2p peak fit parameters for the clean substrate and the two thinnest CoPc film thicknesses related to Figure 2a.

**0.45 nm**	**D1 (Ti^{4+})**<br>Ti-2p$_{3/2}$<br>Ti-2p$_{1/2}$	**0.25 nm**	**D1 (Ti^{4+})**<br>Ti-2p$_{3/2}$<br>Ti-2p$_{1/2}$	**0.0 nm**	**D1 (Ti^{4+})**<br>Ti-2p$_{3/2}$<br>Ti-2p$_{1/2}$
peak position [BE, eV]	459.04<br>464.77		458.86<br>464.59		459.16<br>464.89
LW [eV]	0.33<br>1.53		0.33<br>1.53		0.33<br>1.53
GW [eV]	0.96<br>1.04		0.99<br>1.07		0.96<br>1.05
rel. area [%]	61.3<br>38.7		61.6<br>38.4		61.5<br>38.5

Table S2: O 1s peak fit parameters for the clean substrate and the two thinnest CoPc film thicknesses related to Figure S3a.

0.45 nm	S1<br>O-1	0.25 nm	S1<br>O-1	0.0 nm	S1<br>O-1
peak position [BE, eV]	530.32		530.15		530.45
LW [eV]	0.23		0.23		0.23
GW [eV]	1.20		1.24		1.18

Table S3: Sr 3d peak fit parameters for the clean substrate and the two thinnest CoPc film thicknesses related to Figure S3b.

0.45 nm	D1 (Sr^{2+})<br>Sr-3$d_{5/2}$<br>Sr-3$d_{3/2}$	0.25 nm	D1 (Sr^{2+})<br>Sr-3$d_{5/2}$<br>Sr-3$d_{3/2}$	0.0 nm	D1 (Sr^{2+})<br>Sr-3$d_{5/2}$<br>Sr-3$d_{3/2}$
peak position [BE, eV]	133.73<br>135.46		133.53<br>135.26		133.84<br>135.57
LW [eV]	0.13<br>0.13		0.13<br>0.13		0.13<br>0.13
GW [eV]	1.01<br>1.01		1.04<br>1.04		0.95<br>0.95
rel. area [%]	58.6<br>41.4		58.1<br>41.9		58.2<br>41.8

Substrate spectra and peak fit parameters for $CoPcF_{16}$ on STO(100) prepared by preparation I

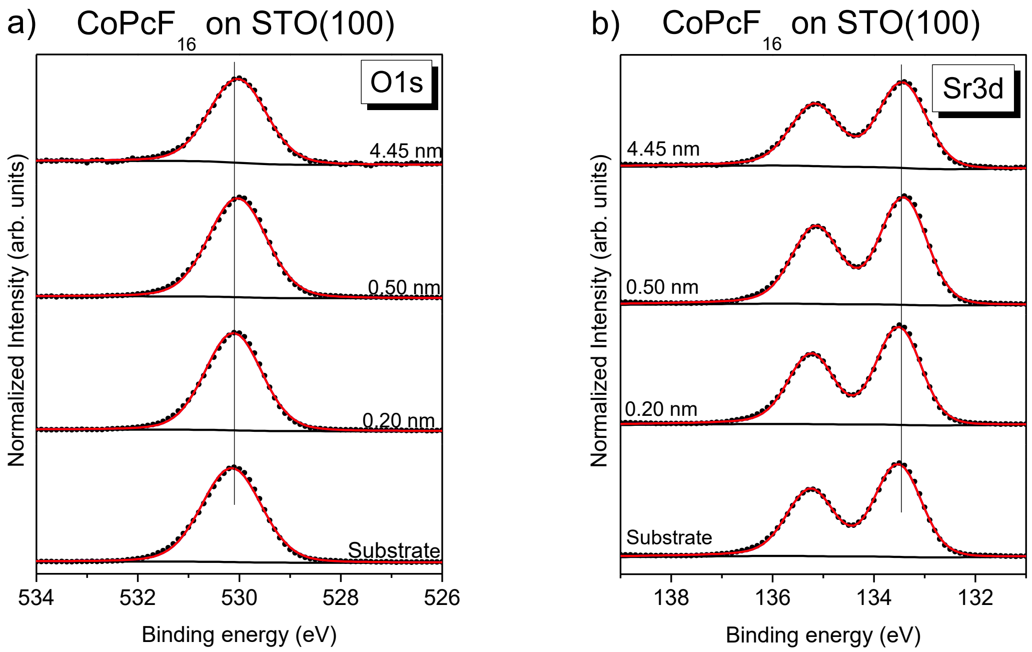

Figure S4: STO(100)-related core level spectra and their development with increasing $CoPcF_{16}$ film thickness. (a) O 1s and (b) Sr 3d. All spectra ca be fitted using a single singlet (O 1s) or doublet (Sr 3d) peak.

Table S4: Ti 2p peak fit parameters for $CoPcF_{16}$ related to Figure 2b.

**0.50 nm**	**D1 (Ti^{4+})**<br>Ti-2p$_{3/2}$<br>Ti-2p$_{1/2}$	**0.20 nm**	**D1 (Ti^{4+})**<br>Ti-2p$_{3/2}$<br>Ti-2p$_{1/2}$	**0.0 nm**	**D1 (Ti^{4+})**<br>Ti-2p$_{3/2}$<br>Ti-2p$_{1/2}$
peak position [BE, eV]	458.78<br>464.50		458.86<br>464.58		458.89<br>464.62
LW [eV]	0.34<br>1.24		0.34<br>1.24		0.34<br>1.24
GW [eV]	0.96<br>1.30		0.91<br>1.25		0.96<br>1.28
rel. area [%]	62.8<br>37.2		62.6<br>37.4		63.1<br>36.9

Table S5: O 1s peak fit parameters for $CoPcF_{16}$ related to Figure S4a.

0.50 nm	S1 O-1	0.20 nm	S1 O-1	0.0 nm	S1 O-1
peak position [BE, eV]	530.03		530.12		530.15
LW [eV]	0.21		0.21		0.21
GW [eV]	1.24		1.20		1.27

Table S6: Sr 3d peak fit parameters for $CoPcF_{16}$ related to Figure S4b.

0.50 nm	D1 (Sr^{2+}) Sr-$3d_{5/2}$ Sr-$3d_{3/2}$	0.20 nm	D1 (Sr^{2+}) Sr-$3d_{5/2}$ Sr-$3d_{3/2}$	0.0 nm	D1 (Sr^{2+}) Sr-$3d_{5/2}$ Sr-$3d_{3/2}$
peak position [BE, eV]	133.42<br>135.14		133.51<br>135.23		133.52<br>135.24
LW [eV]	0.13<br>0.13		0.13<br>0.13		0.13<br>0.13
GW [eV]	1.02<br>1.02		0.97<br>0.97		1.01<br>1.01
Asymmetry	0.02<br>0.025		0.016<br>0.021		0.016<br>0.021
rel. area [%]	58.1<br>41.9		58.2<br>41.8		58.1<br>41.9

Sr 3p background subtraction prior to C 1s peak fit

The measured spectra of the background signal and the two thinnest organic layers (0.25 and 0.45 nm) are compared in Figure S5a. The subtraction of the background signal should reduce the increasing intensity at lower and higher binding energy of the C 1s peak for the organic layers originating from the Sr 3p signal. In the next step the background signal is multiplied by a reducing factor, which is found by variation for the optimum between having no influence on the organic signal and changing the organic signal too much. Then this modified background, which is different for the two organic layers, is subtracted. The result is the blue curve in Figure S5b and Figure S5c, which is then used for the peak fits. The same approach is used for the other organic molecules.

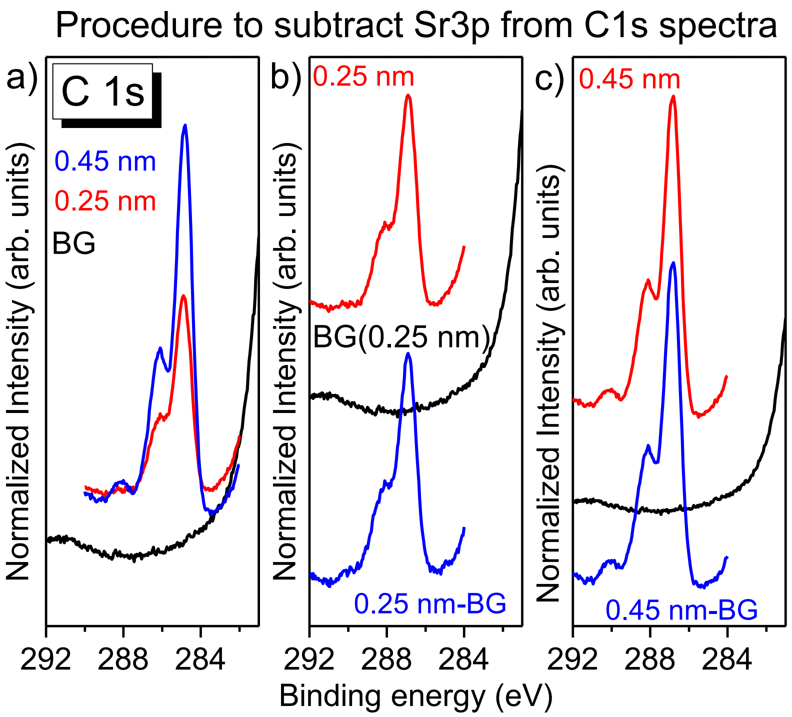

Figure S5: CoPc on STO(100). C 1s core level spectra and their modification for the subtraction of the Sr 3p signal from the C 1s peak for the interface layers of the organic molecules.

N 1s and C 1s peak fit parameters for CoPc on STO(100) prepared by preparation I

Table S7: Peak fit parameters for N 1s peak fit of CoPc for Figure 3a.

**6.90 nm**	**S1** N-1	**S2** S(N-1)	**0.25 nm**	**S1** N-1	**S3** S(N-1)
peak position [BE, eV]	399.05	400.90		399.23	400.77
LW [eV]	0.10	0.10		0.10	0.10
GW [eV]	0.96	0.96		1.10	1.10
rel. area [%]	95.9	4.1		95.8	4.2

Table S8: Peak fit parameters for C 1s spectra of CoPc for Figure 3b.

**6.90 nm**	**S1** C-1	**S2** S(C-1)	**S3** C-2	**S4** S(C-2)	**0.25 nm**	**S1** C-1	**S2** S(C-1)	**S3** C-2	**S4** S(C-2)
peak position [BE, eV]	284.62	286.38	285.94	287.99		284.89	286.71	286.06	288.01
LW [eV]	0.10	0.10	0.10	0.10		0.10	0.10	0.10	0.10
GW [eV]	0.76	0.76	0.76	0.76		0.94	0.94	0.94	0.94
rel. area [%]	69.5	6.7	20.4	3.4		68.7	6.9	22.9	1.5

C 1s peak fit parameters and N 1s spectrum for $CoPcF_{16}$ on STO(100) prepared by preparation I

Table S9: Thickness-dependent C 1s peak fit parameters of $CoPcF_{16}$ evaporated on STO(100) prepared by preparation I for fig. 4a. There are given three tables, one for each thickness: (a) 4.45 nm, (b) 0.50 nm, and c) 0.20 nm.

**a) 4.45 nm**	**S1**<br>C-1	**S2**<br>S(C-1)	**S3**<br>C-2	**S4**<br>S(C-2)	**S5**<br>C-3	**S6**<br>S(C-3)
peak position [BE, eV]	284.83	286.58	285.94	288.01	287.19	288.88
LW [eV]	0.10	0.10	0.10	0.10	0.10	0.10
GW [eV]	0.78	0.78	0.78	0.78	0.78	0.78
rel. area [%]	23.2	1.1	19.0	6.4	44.0	6.3

**b) 0.50 nm**	**S1**<br>C-1	**S2**<br>S(C-1)	**S3**<br>C-2	**S4**<br>S(C-2)	**S5**<br>C-3	**S6**<br>S(C-3)
peak position [BE, eV]	285.04	286.79	285.93	288.02	287.30	289.03
LW [eV]	0.10	0.10	0.10	0.10	0.10	0.10
GW [eV]	1.13	1.13	1.13	1.13	1.13	1.13
rel. area [%]	24.0	1.1	19.7	6.6	42.0	6.6

**c) 0.20 nm**	**S1**<br>C-1	**S2**<br>S(C-1)	**S3**<br>C-2	**S4**<br>S(C-2)	**S5**<br>C-3	**S6**<br>S(C-3)
peak position [BE, eV]	285.20	286.95	285.97	287.94	287.45	289.19
LW [eV]	0.10	0.10	0.10	0.10	0.10	0.10
GW [eV]	1.13	1.13	1.13	1.13	1.13	1.13
rel. area [%]	24.0	1.1	19.7	6.6	42.0	6.6

Table S10: F1s peak fit parameters of $CoPcF_{16}$ as shown in Figure 4b.

**4.45 nm**	**S1** F-1	**S2** S(F-1)	**S3** FE-1	**0.50 nm**	**S1** F-1	**S2** S(F-1)	**S3** FE-1	**0.20 nm**	**S1** F-1	**S2** S(F-1)	**S3** FE-1
peak position [BE, eV]	687.16	688.75	685.14		687.59	689.18	684.59		687.99	689.58	684.71
LW [eV]	0.10	0.10	0.10		0.10	0.10	0.10		0.10	0.10	0.10
GW [eV]	1.29	1.29	1.29		1.53	1.53	1.53		1.54	1.54	1.54
rel. area [%]	91.3	7.9	0.8		83.7	7.3	9.0		76.7	6.7	16.6

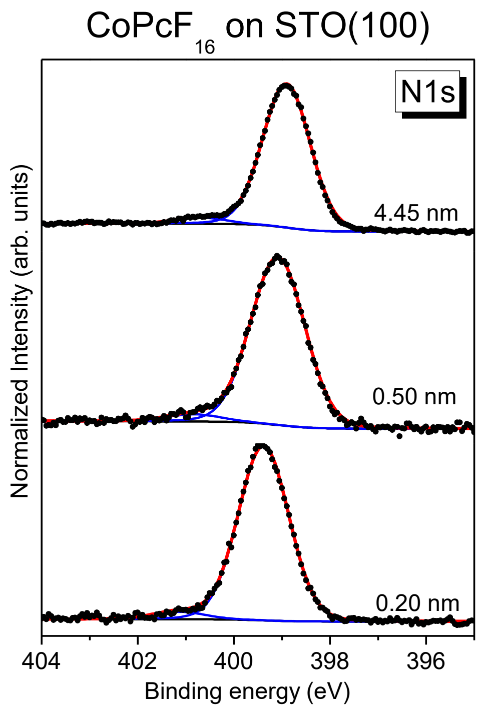

Figure S6: $CoPcF_{16}$ on STO(100). N 1s core level spectra for a thicker film compared to a coverage of a monolayer. No changes of the peak shape were detected.

Table 11: Thickness-dependent N 1s peak fit parameters of $CoPcF_{16}$ evaporated on STO(100) prepared by preparation I for Figure S6.

**4.45 nm**	**S1** N-1	**S2** S(N-1)	**0.50 nm**	**S1** N-1	**S2** S(N-1)	**0.20 nm**	**S1** N-1	**S2** S(N-1)
peak position [BE, eV]	398.91	400.66		399.09	400.84		399.38	401.13
LW [eV]	0.10	0.10		0.10	0.10		0.10	0.10
GW [eV]	1.10	1.10		1.27	1.27		1.18	1.18
rel. area [%]	95.9	4.1		95.9	4.1		95.9	4.1

Binding energy shift of the C 1s C-1 component for the different molecules

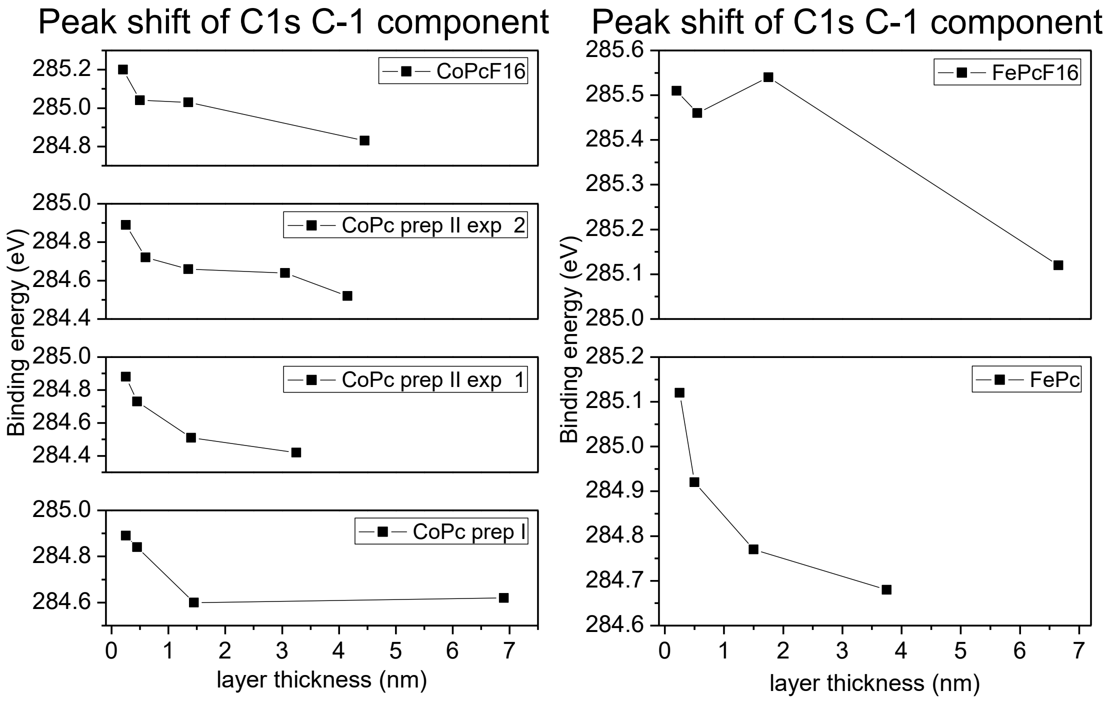

Figure S7: Shifts of the binding energy of the C 1s C-1 component for the different molecules and preparations: $CoPcF_x$ on the left, $FePcF_x$ on the right. It is observable as general trend, that the thickest layer shows the lowest binding energy and the binding energy increases with decreasing film thickness.

Substrate spectra and peak fit parameters for FePc on STO(100) prepared by preparation I

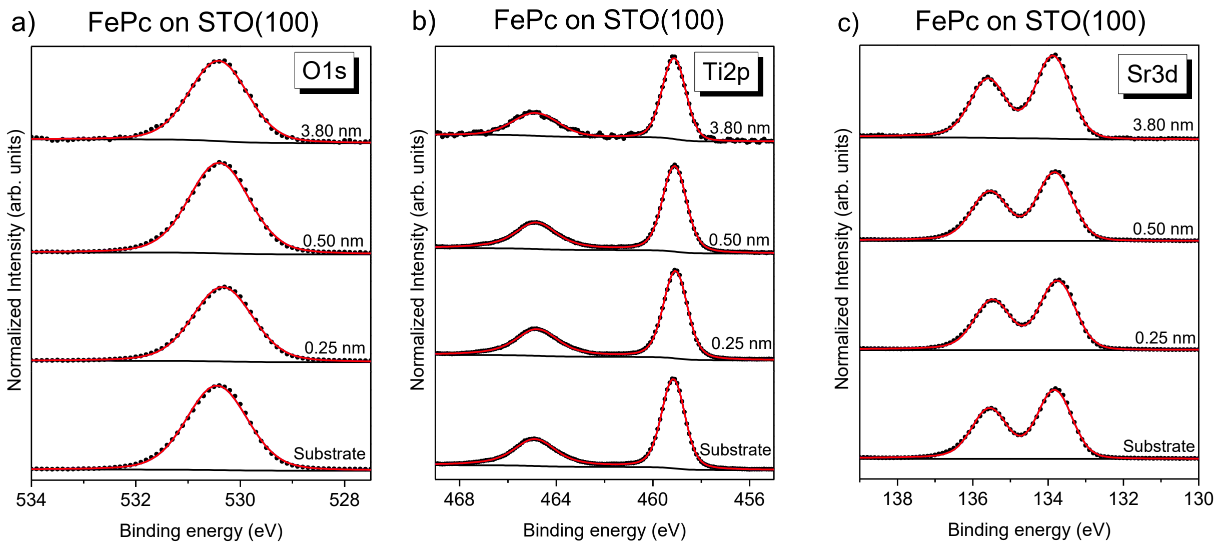

Figure S8: STO(100)-related core level spectra and their development with increasing FePc film thickness. (a) O 1s, (b) Ti 2p, and (c) Sr 3d. All spectra ca be fitted using a single singlet (O 1s) or doublet (Ti 2p, Sr 3d) peak.

Table S12: O 1s peak fit parameters for the clean substrate and the two thinnest FePc film thicknesses related to Figure S8a.

**0.50 nm**	**S1** O-1	**0.25 nm**	**S1** O-1	**0.0 nm**	**S1** O-1
peak position [BE, eV]	530.44		530.34		530.40
LW [eV]	0.31		0.31		0.28
GW [eV]	1.19		1.18		1.22

Table S13. Ti 2p peak fit parameters for the clean substrate and the two thinnest FePc film thicknesses related to Figure S8b.

**0.50 nm**	**D1 (Ti^{4+})**<br>Ti-$2p_{3/2}$<br>Ti-$2p_{1/2}$	**0.25 nm**	**D1 (Ti^{4+})**<br>Ti-$2p_{3/2}$<br>Ti-$2p_{1/2}$	**0.0 nm**	**D1 (Ti^{4+})**<br>Ti-$2p_{3/2}$<br>Ti-$2p_{1/2}$
peak position [BE, eV]	459.11<br>464.83		459.07<br>464.79		459.16<br>464.88
LW [eV]	0.33<br>1.45		0.33<br>1.40		0.32<br>1.38
GW [eV]	0.98<br>1.09		0.98<br>1.21		0.96<br>1.18
rel. area [%]	61.8<br>38.2		62.0<br>38.0		62.0<br>38.0

Table S14: Sr 3d peak fit parameters for the clean substrate and the two thinnest FePc film thicknesses related to Figure S8c.

**0.50 nm**	**D1 (Sr^{2+})**<br>Sr-$3d_{5/2}$<br>Sr-$3d_{3/2}$	**0.25 nm**	**D1 (Sr^{2+})**<br>Sr-$3d_{5/2}$<br>Sr-$3d_{3/2}$	**0.0 nm**	**D1 (Sr^{2+})**<br>Sr-$3d_{5/2}$<br>Sr-$3d_{3/2}$
peak position [BE, eV]	133.81<br>135.53		133.74<br>135.46		133.82<br>135.54
LW [eV]	0.13<br>0.13		0.13<br>0.13		0.13<br>0.13
GW [eV]	1.04<br>1.04		1.03<br>1.03		1.01<br>1.01
rel. area [%]	58.3<br>41.7		58.5<br>41.5		58.0<br>42.0

FePc related spectra and peak fit parameters for the experiment FePc on STO(100) prepared by preparation I

Table S15: Peak fit parameters for N 1s peak fit of FePc, see Figure 5a.

**3.80 nm**	**S1**<br>N-1	**S2**<br>S(N-1)	**0.25 nm**	**S1**<br>N-1	**S3**<br>S(N-1)
peak position [BE, eV]	399.04	400.67		399.36	400.99
LW [eV]	0.11	0.11		0.11	0.11
GW [eV]	0.92	0.92		1.08	1.08
rel. area [%]	95.9	4.1		95.9	4.1

Table S16: Peak fit parameters for C 1s peak fit of FePc, see Figure 5b.

**3.80 nm**	**S1**<br>C-1	**S2**<br>S(C-1)	**S3**<br>C-2	**S4**<br>S(C-2)	**0.25 nm**	**S1**<br>C-1	**S2**<br>S(C-1)	**S3**<br>C-2	**S4**<br>S(C-2)
peak position [BE, eV]	284.68	286.47	285.96	287.96		285.12	286.82	286.18	288.07
LW [eV]	0.10	0.10	0.10	0.10		0.10	0.10	0.10	0.10
GW [eV]	0.78	0.78	0.78	0.78		0.95	0.95	0.95	0.95
rel. area [%]	69.3	6.2	21.2	3.3		66.0	9.2	21.7	3.1

Substrate spectra and peak fit parameters for $FePcF_{16}$ on STO(100) prepared by preparation I

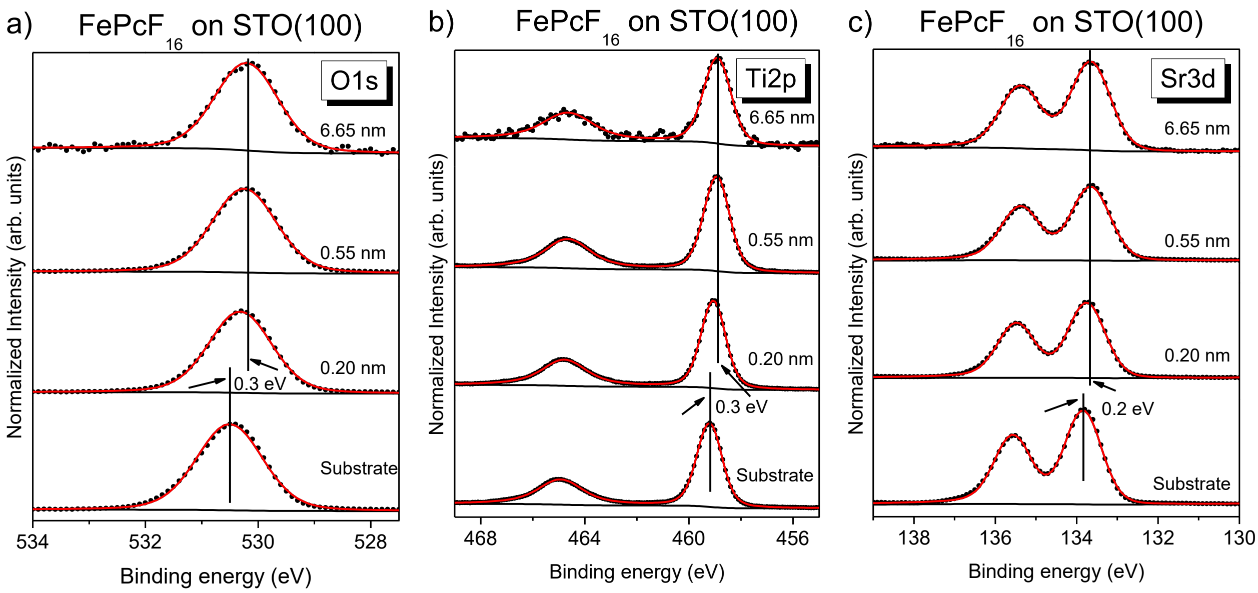

Figure S9: STO(100)-related core level spectra and their development with increasing $FePcF_{16}$ film thickness. (a) O 1s, (b) Ti 2p, and (c) Sr 3d. Small energy shifts may point to doping as a result of the $FePcF_{16}$ adsorption. All spectra ca be fitted using a single singlet (O 1s) or doublet (Ti 2p, Sr 3d) peak.

Table S17: O 1s peak fit parameters for $FePcF_{16}$ related to Figure S9a.

**0.55 nm**	**S1** O-1	**0.20 nm**	**S1** O-1	**0.0 nm**	**S1** O-1
peak position [BE, eV]	530.25		530.32		530.50
LW [eV]	0.29		0.29		0.29
GW [eV]	1.18		1.15		1.22

Table S18: Ti 2p peak fit parameters for $FePcF_{16}$ related to Figure S9b.

**0.55 nm**	**D1 (Ti^{4+})**<br>Ti-$2p_{3/2}$<br>Ti-$2p_{1/2}$	**0.20 nm**	**D1 (Ti^{4+})**<br>Ti-$2p_{3/2}$<br>Ti-$2p_{1/2}$	**0.0 nm**	**D1 (Ti^{4+})**<br>Ti-$2p_{3/2}$<br>Ti-$2p_{1/2}$
peak position [BE, eV]	458.93<br>464.65		459.06<br>464.78		459.21<br>464.93
LW [eV]	0.32<br>1.45		0.32<br>1.45		0.32<br>1.45
GW [eV]	0.99<br>1.14		0.93<br>1.10		0.95<br>1.11
rel. area [%]	61.6<br>38.4		61.7<br>38.3		61.7<br>38.3

Table S19: Sr 3d peak fit parameters for $FePcF_{16}$ related to Figure S9c.

**0.55 nm**	**D1 (Sr^{2+})**<br>Sr-$3d_{5/2}$<br>Sr-$3d_{3/2}$	**0.20 nm**	**D1 (Sr^{2+})**<br>Sr-$3d_{5/2}$<br>Sr-$3d_{3/2}$	**0.0 nm**	**D1 (Sr^{2+})**<br>Sr-$3d_{5/2}$<br>Sr-$3d_{3/2}$
peak position [BE, eV]	133.65<br>135.37		133.75<br>135.47		133.83<br>135.55
LW [eV]	0.13<br>0.13		0.13<br>0.13		0.13<br>0.13
GW [eV]	1.04<br>1.04		0.99<br>0.99		0.98<br>0.98
Asymmetry	0.023<br>0.029		0.023<br>0.029		0.023<br>0.029
rel. area [%]	58.3<br>41.7		58.3<br>41.7		58.0<br>42.0

Table S20: F1s peak fit parameters of $FePcF_{16}$ as shown in Figure 6a.

**6.65 nm**	**S1**<br>F-1	**S2**<br>S(F-1)
peak position [BE, eV]	687.43	688.99
LW [eV]	0.23	0.23
GW [eV]	1.19	1.19
rel. area [%]	93.9	6.1

**0.55 nm**	**S1**<br>F-1	**S2**<br>S(F-1)	**S3**<br>FE-1	**S4**<br>S(FE-1)	**0.20 nm**	**S1**<br>F-1	**S2**<br>S(F-1)	**S3**<br>F-2	**S4**<br>S(FE-1)
	687.94	689.54	684.58	686.28		688.21	689.81	684.71	686.41
	0.14	0.14	0.24	0.24		0.14	0.14	0.24	0.24
	1.40	1.40	1.84	1.84		1.47	1.47	1.79	1.79
	87.7	8.2	3.8	0.3		84.4	7.9	7.1	0.6

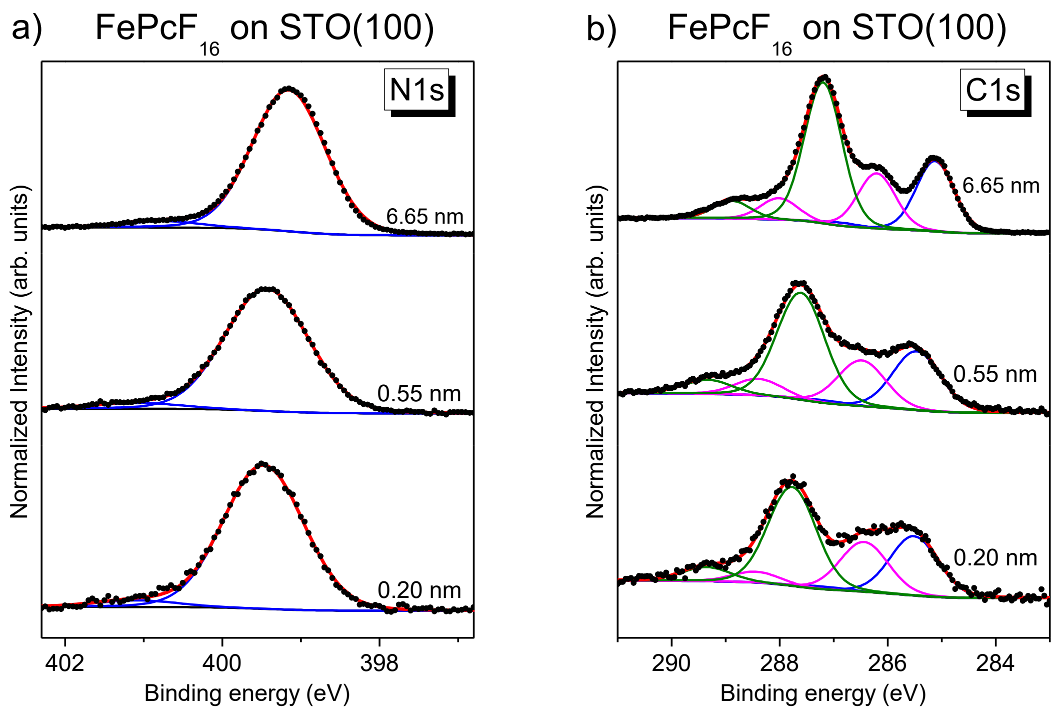

Figure S10: $FePcF_{16}$ on STO(100). Development of core level spectra with increasing film thickness: (a) N 1s and (b) C 1s.

Table 21: Thickness-dependent N 1s peak fit parameters of $FePcF_{16}$ evaporated on STO(100) prepared by preparation I for Figure S10a.

**6.65 nm**	**S1** N-1	**S2** S(N-1)	**0.55 nm**	**S1** N-1	**S2** S(N-1)	**0.20 nm**	**S1** N-1	**S2** S(N-1)
peak position [BE, eV]	399.15	400.75		399.43	401.03		399.48	401.08
LW [eV]	0.10	0.10		0.10	0.10		0.10	0.10
GW [eV]	1.04	1.04		1.20	1.20		1.15	1.15
rel. area [%]	95.6	4.4		95.6	4.4		95.6	4.4

Table S22: Thickness-dependent C 1s peak fit parameters of $FePcF_{16}$ evaporated on STO(100) prepared by preparation I for Figure S10b. There are given three tables, one for each thickness: (a) 6.65 nm, (b) 0.55 nm, and (c) 0.20 nm.

**a) 6.65 nm**	**S1**<br>C-1	**S2**<br>S(C-1)	**S3**<br>C-2	**S4**<br>S(C-2)	**S5**<br>C-3	**S6**<br>S(C-3)
peak position [BE, eV]	285.12	286.87	286.20	288.01	287.19	288.88
LW [eV]	0.10	0.10	0.10	0.10	0.10	0.10
GW [eV]	0.76	0.76	0.76	0.76	0.76	0.76
rel. area [%]	22.8	1.0	17.8	6.9	45.7	5.8

**b) 0.55 nm**	**S1**<br>C-1	**S2**<br>S(C-1)	**S3**<br>C-2	**S4**<br>S(C-2)	**S5**<br>C-3	**S6**<br>S(C-3)
peak position [BE, eV]	285.46	287.21	286.48	288.39	287.60	289.32
LW [eV]	0.11	0.11	0.11	0.11	0.11	0.11
GW [eV]	0.98	0.98	0.98	0.98	0.98	0.98
rel. area [%]	24.1	1.1	18.7	6.7	43.7	5.7

**c) 0.20 nm**	**S1**<br>C-1	**S2**<br>S(C-1)	**S3**<br>C-2	**S4**<br>S(C-2)	**S5**<br>C-3	**S6**<br>S(C-3)
peak position [BE, eV]	285.51	287.26	286.43	288.44	287.77	289.35
LW [eV]	0.11	0.11	0.11	0.11	0.11	0.11
GW [eV]	0.99	0.99	0.99	0.99	0.99	0.99
rel. area [%]	25.2	1.2	21.1	4.6	42.0	5.9

Substrate spectra and peak fit parameters for STO(100) prepared by preparation II

1. Substrate peaks and their development during experiment 1

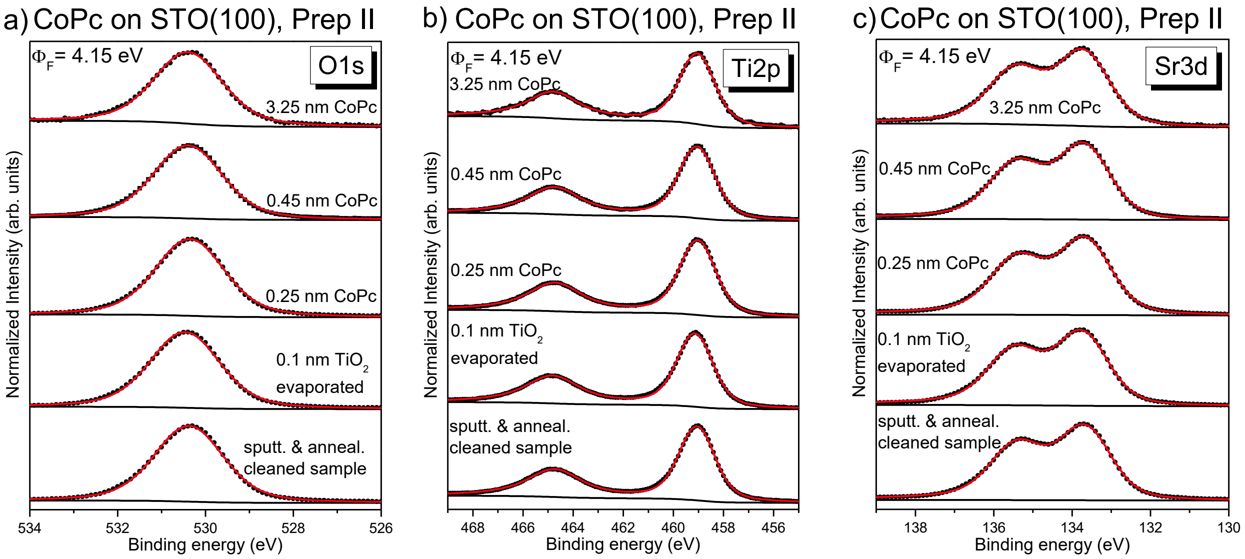

Figure S11: Substrate spectra of experiment 1 from the pure substrate to a thick film of CoPc: (a) O 1s, (b) Ti 2p, and (c) Sr 3d.

Table S23: Peak fit parameters of O 1s in experiment 1 (Φ_F = 4.15 eV) of CoPc evaporated on STO(100) prepared by preparation II.

**0.45 nm**	**S1** O-1	**0.25 nm**	**S1** O-1	**0.0 nm (TiO_2 evap.)**	**S1** O-1	**0.0 nm (Sp. & An.)**	**S1** O-1
peak position [BE, eV]	530.40		530.35		530.47		530.38
LW [eV]	0.65		0.65		0.65		0.65
GW [eV]	1.49		1.49		1.51		1.49

Table S24: Peak fit parameters of Ti 2p in experiment 1 (Φ_F = 4.15 eV) of CoPc evaporated on STO(100) prepared by preparation II.

**0.45 nm**	**D1 (Ti^{4+})** Ti-2p$_{3/2}$ Ti-2p$_{1/2}$	**0.25 nm**	**D1 (Ti^{4+})** Ti-2p$_{3/2}$ Ti-2p$_{1/2}$	**0.0 nm (TiO_2 evap.)**	**D1 (Ti^{4+})** Ti-2p$_{3/2}$ Ti-2p$_{1/2}$	**0.0 nm (Sp. & An.)**	**D1 (Ti^{4+})** Ti-2p$_{3/2}$ Ti-2p$_{1/2}$
peak position [BE, eV]	459.06<br>464.75		459.03<br>464.72		459.14<br>464.82		459.06<br>464.75
LW [eV]	0.65<br>2.26		0.65<br>2.26		0.65<br>2.26		0.65<br>2.26
GW [eV]	1.34<br>0.90		1.34<br>0.90		1.33<br>0.96		1.33<br>1.00
rel. area [%]	60.7<br>39.3		60.3<br>39.7		60.5<br>39.5		60.4<br>39.6

Table S25: Peak fit parameters of Sr 3d in experiment 1 (Φ_F = 4.15 eV) of CoPc evaporated on STO(100) prepared by preparation II.

**0.45 nm**	**D1 (Sr^{2+})** Sr-3d$_{5/2}$ Sr-3d$_{3/2}$	**0.25 nm**	**D1 (Sr^{2+})** Sr-3d$_{5/2}$ Sr-3d$_{3/2}$	**0.0 nm (TiO_2 evap.)**	**D1 (Sr^{2+})** Sr-3d$_{5/2}$ Sr-3d$_{3/2}$	**0.0 nm (Sp. & An.)**	**D1 (Sr^{2+})** Sr-3d$_{5/2}$ Sr-3d$_{3/2}$
peak position [BE, eV]	133.68<br>135.41		133.65<br>135.38		133.73<br>135.46		133.66<br>135.39
LW [eV]	0.47<br>0.47		0.47<br>0.47		0.47<br>0.47		0.47<br>0.47
GW [eV]	1.31<br>1.31		1.31<br>1.31		1.30<br>1.30		1.30<br>1.30
Asymmetry	0.027<br>0.039		0.027<br>0.039		0.027<br>0.039		0.027<br>0.039
rel. area [%]	57.5<br>42.5		57.5<br>42.5		57.2<br>42.8		57.1<br>42.9

2. Substrate peaks and their development during experiment 2

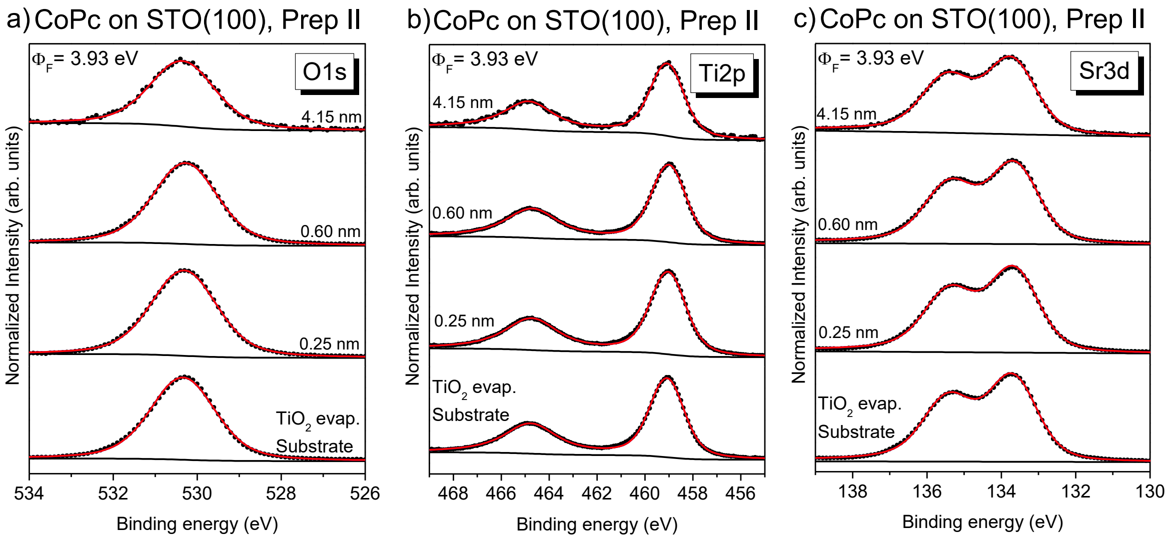

Figure S12: Substrate spectra of experiment 2 from the pure substrate to a thick film of CoPc on STO(100) prepared by preparation II: (a) O 1s, (b) Ti 2p, and (c) Sr 3d.

Table 26: Peak fit parameters of O 1s in experiment 2 of CoPc evaporated on STO(100) prepared by preparation II.

**0.6 nm**	**S1** O-1	**0.25 nm**	**S1** O-1	**0.0 nm (TiO$_2$ evap.)**	**S1** O-1
peak position [BE, eV]	530.28		530.31		530.34
LW [eV]	0.65		0.65		0.65
GW [eV]	1.43		1.47		1.45

Table 27: Peak fit parameters of Ti 2p in experiment 2 of CoPc evaporated on STO(100) prepared by preparation II.

0.6 nm	D1 (Ti^{4+}) Ti-2$p_{3/2}$ Ti-2$p_{1/2}$	0.25 nm	D1 (Ti^{4+}) Ti-2$p_{3/2}$ Ti-2$p_{1/2}$	0.0 nm (TiO_2 evap.)	D1 (Ti^{4+}) Ti-2$p_{3/2}$ Ti-2$p_{1/2}$
peak position [BE, eV]	459.01<br>464.72		459.04<br>464.73		459.09<br>464.75
LW [eV]	0.65<br>2.26		0.65<br>2.26		0.65<br>2.26
GW [eV]	1.34<br>0.98		1.38<br>1.04		1.33<br>0.97
rel. area [%]	60.4<br>39.6		60.2<br>39.8		60.1<br>39.9

Table S28. Peak fit parameters of Sr 3d in experiment 2 of CoPc evaporated on a STO(100) prepared by preparation II.

0.6 nm	D1 (Sr^{2+}) Sr-3$d_{5/2}$ Sr-3$d_{3/2}$	0.25 nm	D1 (Sr^{2+}) Sr-3$d_{5/2}$ Sr-3$d_{3/2}$	0.0 nm (TiO_2 evap.)	D1 (Sr^{2+}) Sr-3$d_{5/2}$ Sr-3$d_{3/2}$
peak position [BE, eV]	133.64<br>135.37		133.65<br>135.38		133.68<br>135.41
LW [eV]	0.47<br>0.47		0.47<br>0.47		0.47<br>0.47
GW [eV]	1.29<br>1.29		1.29<br>1.29		1.28<br>1.28
Asymmetry	0.027<br>0.039		0.027<br>0.039		0.027<br>0.039
rel. area [%]	57.7<br>42.3		58.2<br>41.8		57.7<br>42.3

3. Organic peaks (C 1s and N 1s) of experiment 1

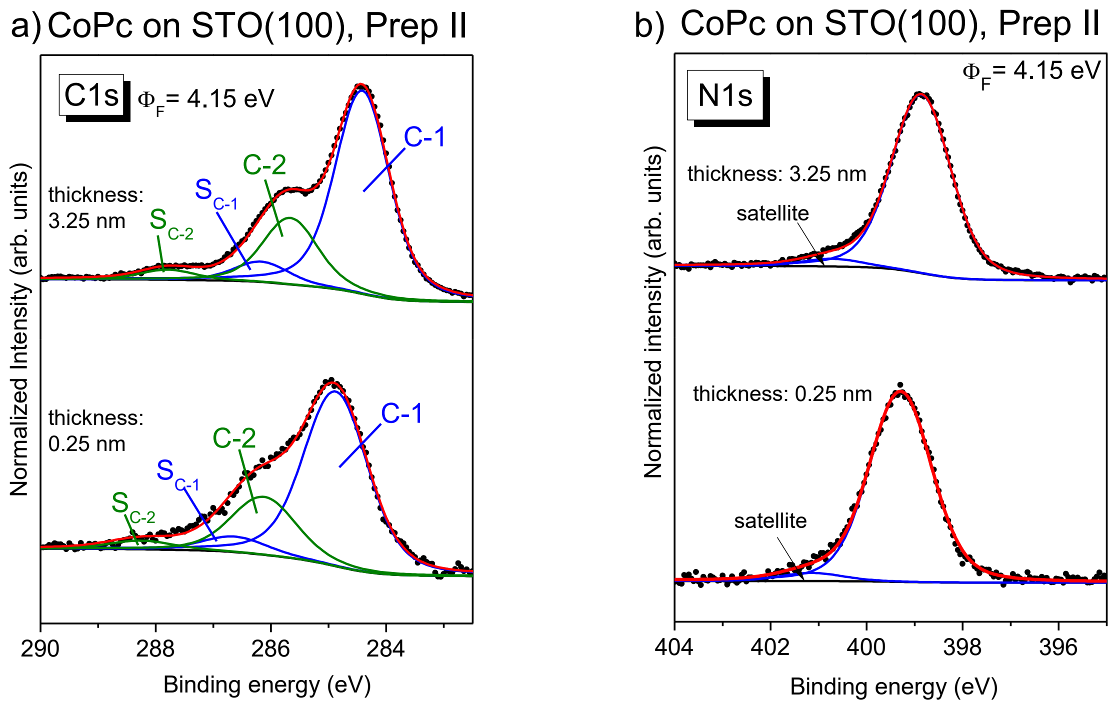

Figure S13: Organic spectra of thinnest and thickest film of experiment 1 of CoPc evaporated on STO(100) prepared by preparation II: (a) C 1s and (b) N 1s.

Table S29: Peak fit parameters of C 1s in experiment 1 of CoPc evaporated on STO(100) prepared by preparation II. The different CoPc layers are given in three tables: (a) 3.25 nm CoPc, (b) 0.45 nm CoPc, and (c) 0.25 nm CoPc.

**a) 3.25 nm**	**S1**<br>C-1	**S2**<br>S(C-1)	**S3**<br>C-2	**S4**<br>S(C-2)
peak position [BE, eV]	284.42	286.18	285.67	287.79
LW [eV]	0.42	0.42	0.42	0.42
GW [eV]	0.87	0.87	0.87	0.87
rel. area [%]	67.7	6.8	22.2	3.4

**b) 0.45 nm**	**S1**<br>C-1	**S2**<br>S(C-1)	**S3**<br>C-2	**S4**<br>S(C-2)
peak position [BE, eV]	284.73	286.49	285.98	288.10
LW [eV]	0.42	0.42	0.42	0.42
GW [eV]	1.04	1.04	1.04	1.04
rel. area [%]	67.3	6.7	22.6	3.4

**c) 0.25 nm**	**S1**<br>C-1	**S2**<br>S(C-1)	**S3**<br>C-2	**S4**<br>S(C-2)
peak position [BE, eV]	284.88	286.64	286.13	288.25
LW [eV]	0.42	0.42	0.42	0.42
GW [eV]	1.08	1.08	1.08	1.08
rel. area [%]	67.9	6.1	22.7	3.3

Table S30: Peak fit parameters of N 1s in experiment 1 of CoPc evaporated on STO(100) prepared by preparation II.

**3.25 nm**	**S1**<br>N-1	**S2**<br>S(N-1)	**0.45 nm**	**S1**<br>N-1	**S2**<br>S(N-1)	**0.25 nm**	**S1**<br>N-1	**S2**<br>S(N-1)
peak position [BE, eV]	398.86	400.71		399.12	400.97		399.30	401.15
LW [eV]	0.40	0.40		0.40	0.40		0.4	0.4
GW [eV]	1.23	1.23		1.30	1.30		1.31	1.31
rel. area [%]	95.9	4.1		95.9	4.1		95.9	4.1

4. Organic peaks (C 1s and N 1s) of experiment 2

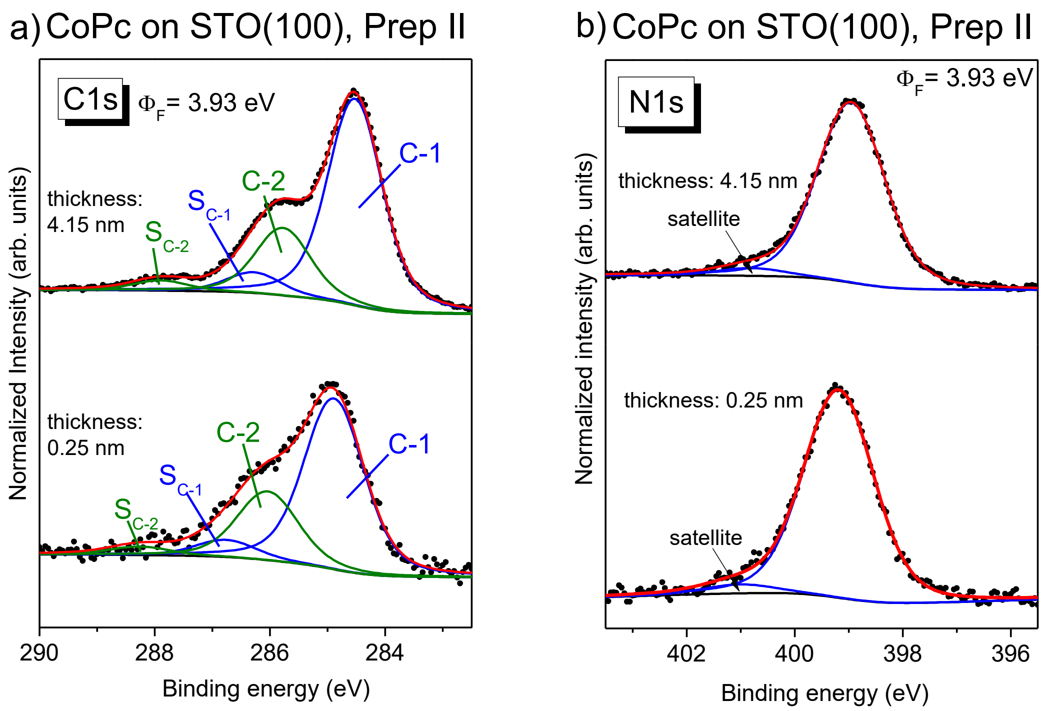

Figure S14: Organic spectra of thinnest and thickest film of experiment 2 of CoPc evaporated on STO(100) in experiment 2: (a) C 1s and (b) N 1s.

Table S31: Peak fit parameters of C 1s in experiment 2 of CoPc evaporated on STO(100) prepared by preparation II. The different CoPc layers are given in three tables: (a) 4.15 nm CoPc, (b) 0.60 nm CoPc and (c) 0.25 nm CoPc.

**a) 4.15 nm**	**S1**<br>C-1	**S2**<br>S(C-1)	**S3**<br>C-2	**S4**<br>S(C-2)
peak position [BE, eV]	284.52	286.28	285.77	287.89
LW [eV]	0.42	0.42	0.42	0.42
GW [eV]	0.88	0.88	0.88	0.88
rel. area [%]	67.7	6.8	22.2	3.3

**b) 0.60 nm**	**S1**<br>C-1	**S2**<br>S(C-1)	**S3**<br>C-2	**S4**<br>S(C-2)
peak position [BE, eV]	284.72	286.62	285.93	288.09
LW [eV]	0.42	0.42	0.42	0.42
GW [eV]	0.99	0.99	0.99	0.99
rel. area [%]	65.2	6.6	25.0	3.2

**c) 0.25 nm**	**S1**<br>C-1	**S2**<br>S(C-1)	**S3**<br>C-2	**S4**<br>S(C-2)
peak position [BE, eV]	284.89	286.76	286.04	288.26
LW [eV]	0.42	0.42	0.42	0.42
GW [eV]	0.99	0.99	0.99	0.99
rel. area [%]	64.3	6.5	26.0	3.2

Table S32: Peak fit parameters of N 1s in the experiment 2 of CoPc evaporated on STO(100) prepared by preparation II.

**4.15 nm**	**S1**<br>N-1	**S2**<br>S(N-1)	**0.60 nm**	**S1**<br>N-1	**S2**<br>S(N-1)	**0.25 nm**	**S1**<br>N-1	**S2**<br>S(N-1)
peak position [BE, eV]	398.95	400.80		399.05	400.90		399.18	401.03
LW [eV]	0.40	0.40		0.40	0.40		0.4	0.4
GW [eV]	1.24	1.24		1.30	1.30		1.31	1.31
rel. area [%]	95.9	4.1		95.9	4.1		95.9	4.1

Co2p spectra of CoPc on STO prepared by preparation II

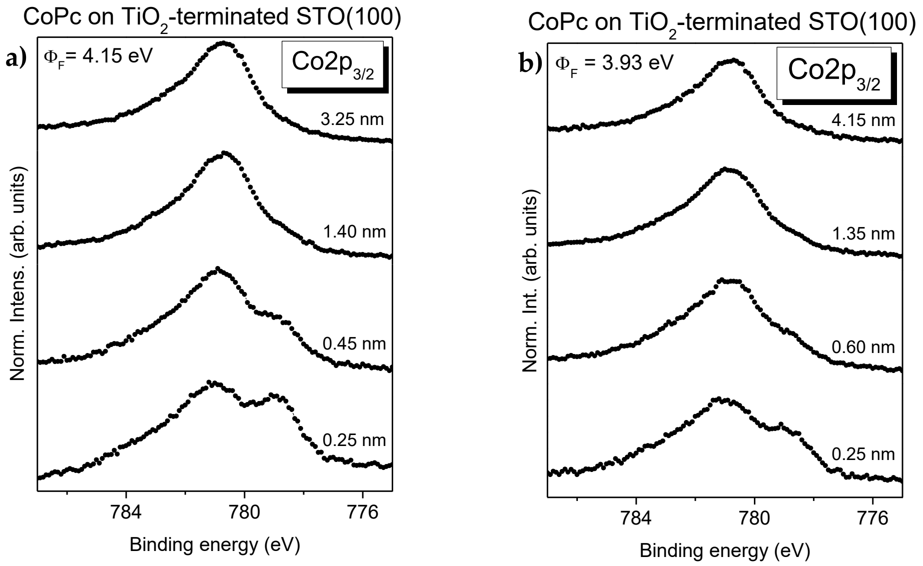

Figure S15: Comparing $Co2p_{3/2}$ spectra from experiment 1 ($\Phi_F = 4.15$ eV) and 2 ($\Phi_F = 3.93$ eV) of CoPc evaporated on STO prepared by preparation II.

References

1. Raisch, C.; Chassé, T.; Langheinrich, C.; Chassé, A. *J. Appl. Phys.* **2012,** *112,* 073505. doi:10.1016/S1389-5567(03)00026-1
2. Chassé, A.; Chassé, T. *J. Phys. Soc. Jpn.* **2018,** *87,* 061006. doi:10.1002/cphc.200700177
